# iPSC reprogramming-mediated aneuploidy correction in autosomal trisomy syndromes

**DOI:** 10.1371/journal.pone.0264965

**Published:** 2022-03-10

**Authors:** Silvia Natsuko Akutsu, Tatsuo Miyamoto, Daiju Oba, Keita Tomioka, Hiroshi Ochiai, Hirofumi Ohashi, Shinya Matsuura

**Affiliations:** 1 Department of Genetics and Cell Biology, Research Institute for Radiation Biology and Medicine, Hiroshima University, Hiroshima, Japan; 2 Department of Genetics, Saitama Children’s Medical Center, Saitama, Japan; 3 Department of Pediatrics, Graduate School of Biomedical and Health Sciences, Hiroshima University, Hiroshima, Japan; 4 Graduate School of Integrated Sciences for Life, Hiroshima University, Higashi-Hiroshima, Japan; University of Bonn, Institute of Experimental Hematology and Transfusion Medicine, GERMANY

## Abstract

Trisomy 21, 18, and 13 are the major autosomal aneuploidy disorders in humans. They are mostly derived from chromosome non-disjunction in maternal meiosis, and the extra trisomic chromosome can cause several congenital malformations. Various genes on the trisomic chromosomes are intricately involved in the development of disease, and fundamental treatments have not yet been established. However, chromosome therapy has been developed to correct the extra chromosome in cultured patient cells, and it was recently reported that during reprogramming into iPSCs, fibroblasts from a Down syndrome patient lost the extra chromosome 21 due to a phenomenon called trisomy-biased chromosome loss. To gain preliminary insights into the underlying mechanism of trisomy rescue during the early stages of reprogramming, we reprogrammed skin fibroblasts from patients with trisomy syndromes 21, 18, 13, and 9 to iPSC, and evaluated the genomes of the individual iPSC colonies by molecular cytogenetic techniques. We report the spontaneous correction from trisomy to disomy upon cell reprogramming in at least one cell line examined from each of the trisomy syndromes, and three possible combinations of chromosomes were selected in the isogenic trisomy-rescued iPSC clones. Single nucleotide polymorphism analysis showed that the trisomy-rescued clones exhibited either heterodisomy or segmental uniparental isodisomy, ruling out the possibility that two trisomic chromosomes were lost simultaneously and the remaining one was duplicated, suggesting instead that one trisomic chromosome was lost to generate disomic cells. These results demonstrated that trisomy rescue may be a phenomenon with random loss of the extra chromosome and subsequent selection for disomic iPSCs, which is analogous to the karyotype correction in early preimplantation embryos. Our finding is relevant for elucidating the mechanisms of autonomous karyotype correction and future application in basic and clinical research on aneuploidy disorders.

## Introduction

Non-disjunction of chromosomes during cell divisions causes aneuploidy in human embryos. Aneuploidy can occur for every chromosome, but most of them are rarely compatible with life. Among autosomal aneuploid embryos, three types of trisomy are live born: trisomy 21 (Down syndrome), trisomy 18 (Edwards syndrome), and trisomy 13 (Patau syndrome). Down syndrome (DS) is the most frequent autosomal trisomy, occurring in approximately 1 in 750 live births. DS patients show a characteristic facial appearance, including a flat nose, large tongue and upward slant to the eye, intellectual disability, intrauterine growth restriction and an increased risk of developing leukemia **[1]**. Edwards syndrome occurs in 1 in 6,000–8,000 live births, and its features include intrauterine growth restriction, microcephaly, micrognathia, hypertonia, small pelvis, clenched fists with the second and fifth fingers overlapping, and shortened life expectancy **[2]**. Patau syndrome is the third most common trisomy, occurring in 1 in 20,000 live births. Its features results from an early developmental defect, leading to midline malformations, including absence of the olfactory nerve and bulb, deafness, holoprosencephaly, and midline cleft lip and palate, which gives a shortened life expectancy [3,4].

Chromosome therapy has been developed to correct an excess trisomic chromosome in cultured patient cells. Li et al **[5]** demonstrated that the use of a conventional gene targeting-mediated dual drug selection cassette to knock-in a TKneo transgene into the APP gene locus of the extra chromosome 21 in induced pluripotent stem cells (iPSC) from DS patients, followed by positive and negative drug selection, allowed correction of aneuploidy by spontaneous loss of one chromosome 21. Jiang et al **[6]** introduced a doxycycline-inducible promoter-driven XIST gene into one copy of chromosome 21 by genome editing technology in DS patient iPSCs. The XIST gene induced stable heterochromatin modification to form a “chromosome 21 Barr body”, which inactivated one extra chromosome 21. Hirota et al **[7]** reported that during reprogramming into iPSCs, fibroblasts from DS lost the extra chromosome 21 through a phenomenon described as “trisomy-biased chromosome loss”, generating euploid iPSCs. Following this, another additional phenomenon was described as “autonomous trisomic rescue”, in which trisomy 21 was spontaneously corrected to disomy in late passages of DS iPSCs **[8]**. These studies imply that trisomy 21 can be corrected to disomy by cell reprogramming.

To gain preliminary insights into the underlying mechanism of trisomy rescue during the early stages of reprogramming, we reprogrammed skin fibroblasts from patients with trisomy syndromes 21, 18, 13, and 9 using episomal vectors, and the independent iPSC colonies that appeared were immediately analyzed using interphase fluorescence in situ hybridization (FISH), karyotype analysis, and single nucleotide polymorphism (SNP) array to minimize the effects by passaging of cell culture.

We found that trisomy rescue occurred in at least one cell line with each of the trisomy syndromes examined, suggesting that trisomy rescue upon reprogramming may be a general phenomenon. Moreover, we found three different combinations of chromosomes in the isogenic trisomy-rescued iPSC clones, and these were classified into two groups: segmental uniparental isodisomy (both retained chromosomes inherited from the same parent) and heterodisomy, suggesting that one trisomic chromosome was lost to generate disomic cells. Taking the results together, we then discuss possible mechanisms for trisomy rescue upon cell reprogramming.

## Results

### Trisomy 13 rescue to disomy

**Fig 1:**
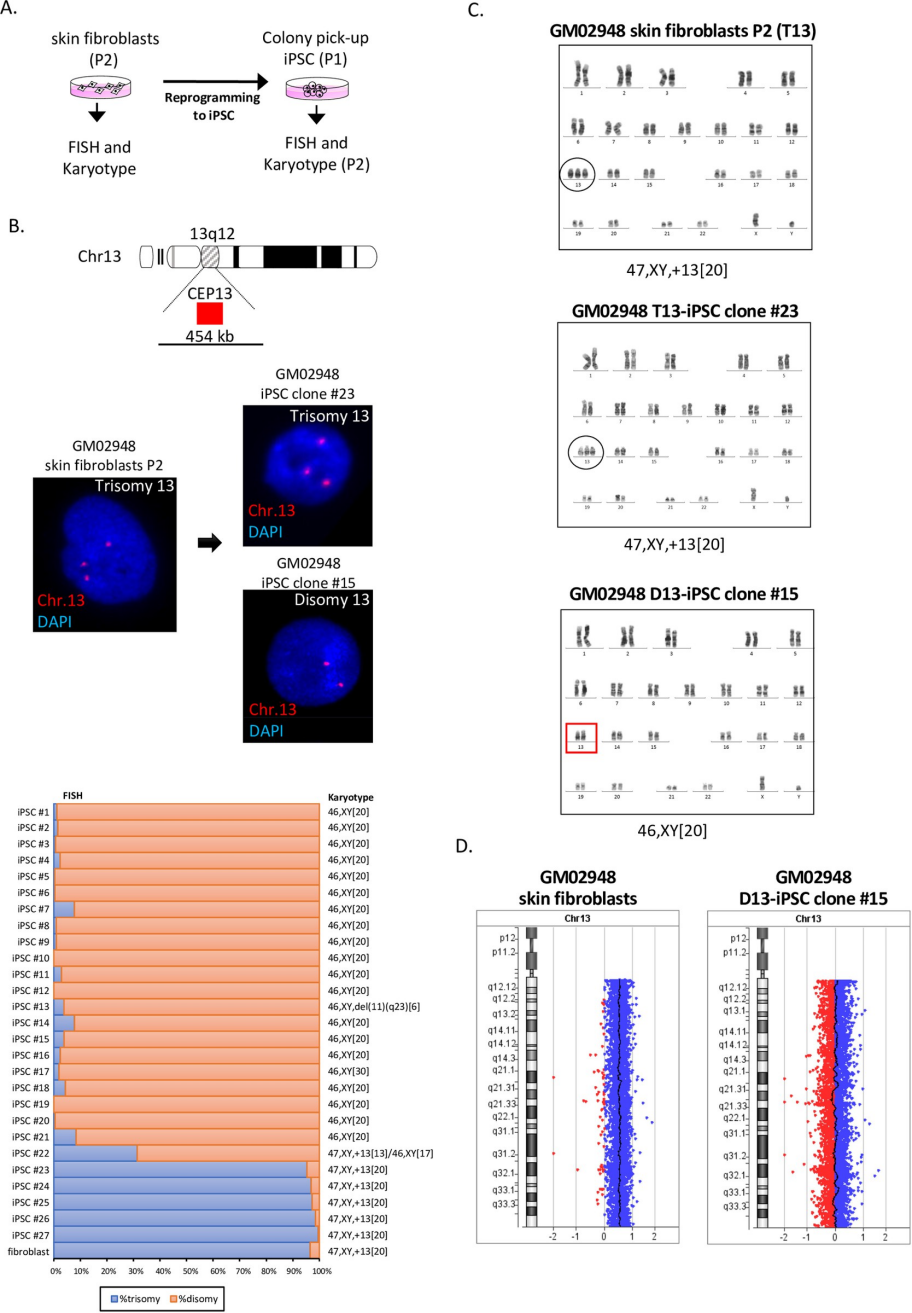
Trisomy 13 of GM02948 skin fibroblasts derived from a Patau syndrome patient was rescued to disomy by cell reprogramming. (A) Scheme of an experimental procedure. Skin fibroblast lines at passage 2 were reprogrammed and the resultant iPSC colonies were picked up (passage 1) and examined for trisomy rescue at passage 2 by interphase fluorescence in situ hybridization (FISH) and karyotype analysis. (B) Interphase FISH analysis using chromosome 13 centromere enumeration probe (CEP13) Orange-Red. DAPI staining shows nucleus (blue). Both the skin fibroblast line and the resultant iPSC clone #23 show trisomy 13 (three red signals), while the iPSC clone #15 shows disomy 13 (two red signals). The graph below shows the interphase FISH results of trisomy and disomy in the skin fibroblasts and the isogenic iPSCs, with the karyotype results on the right. (C) Both skin fibroblasts and the iPSC clone #23 show a male karyotype with trisomy 13 in 20 out of 20 metaphases, while the iPSC clone #15 was trisomy-rescued to show a normal male karyotype in 20 out of 20 metaphases. (D) Array CGH profile for chromosome 13 showing an increased copy number of 0.5 in the fibroblasts, indicating a gain of chromosome 13, while a copy number of zero was observed in iPSC clone #15, indicating no imbalance for chromosome 13 content.

We obtained skin fibroblasts lines from three unrelated patients with trisomy 13 (GM02948, GM03330, and GM00526). Karyotype analysis revealed that all three fibroblast lines exhibited trisomy 13 in 20 out of 20 metaphases (47,XY,+13[20]). We reprogrammed these fibroblast lines at P2 using episomal vectors and picked up the independent iPSC colonies (P1). All the iPSC clones established were morphologically indistinguishable from wild type and expressed the stem-cell markers Oct4, SSEA4, Sox2 and TRA-1-60 (S1 Fig). The iPSC clones at P2 were examined for trisomy rescue by interphase FISH and karyotype analysis (Fig 1A).

Twenty-seven iPSC clones were generated from skin fibroblasts GM02948. Interphase FISH revealed that 22 iPSC clones (81%) had disomy 13 while 6 iPSC clones (19%) remained as trisomy 13 (Fig 1B and 1C). Karyotype analysis indicated that the trisomy-rescued iPSC clones showed 46,XY in 20 of 20 metaphases analyzed (46,XY[20]), except for the clone iPSC#22. This clone showed trisomy 13 mosaicism, as trisomy 13 was detected in 169 of 538 cells (31.4%) by interphase FISH and in 13 of 30 cells (43.3%) by karyotype analysis (Fig 1B, Table 1). Chromosome 11q aberration was identified in one trisomy-rescued clone, iPSC#13. We also performed array comparative genomic hybridization analysis to confirm trisomy 13 in GM02948 skin fibroblasts and chromosome 13 diploidy in the isogenic iPSC#15 clone (Fig 1D). These results suggested that cellular reprogramming of GM02948 fibroblasts rescues trisomy 13 to disomy.

**Table 1:** Numerical alteration for chromosomes in the cells with autosomal trisomies after cell reprogramming*.

		Number of non-rescued iPSC clones		Number of trisomy-rescued iPSC clones				
Subject	Cell line	w/o chr neumerical abnormalities	with chr neumerical abnormalities	w/o chr neumerical abnormalities	with chr neumerical abnormalities	Trisomy rescue efficiency (%)	Number of polyploid clones	Total number of clones
Trisomy 13	GM02948	5	-	22^f^	-	81.5	-	**27**
	GM03330	10	3^a^	9	-	40.9	-	**22**
	GM00526	16	-	5	-	23.8	-	**21**
Trisomy 21	GM02767	21	2^b^	1	-	4.2	-	**24**
	GM04616	10	-	-	-	0.0	-	**10**
Trisomy 18	GM03538	27	1^c^	4	-	12.5	-	**32**
	GM00734	19	-	1^g^	-	5.0	1	**21**
Trisomy 9	GM09286	10	1^d^	14	1^e^	57.7	7	**33**
		**118**	**7**	**56**	**1**	** **	**8**	**190**

^a^Trisomies 1, 20, and X.

^b^Trisomy 1.

^c^Trisomy 20.

^d^Trisomies 1 and 6; Monosomy 4.

^e^Trisomy 11; Monosomies 10 and 13.

^f^One clone had mosaic trisomy.

^g^A clone with mosaic trisomy.

*Totals show in bold, and structural abnormalities are not included in the table.

**Fig 2:**
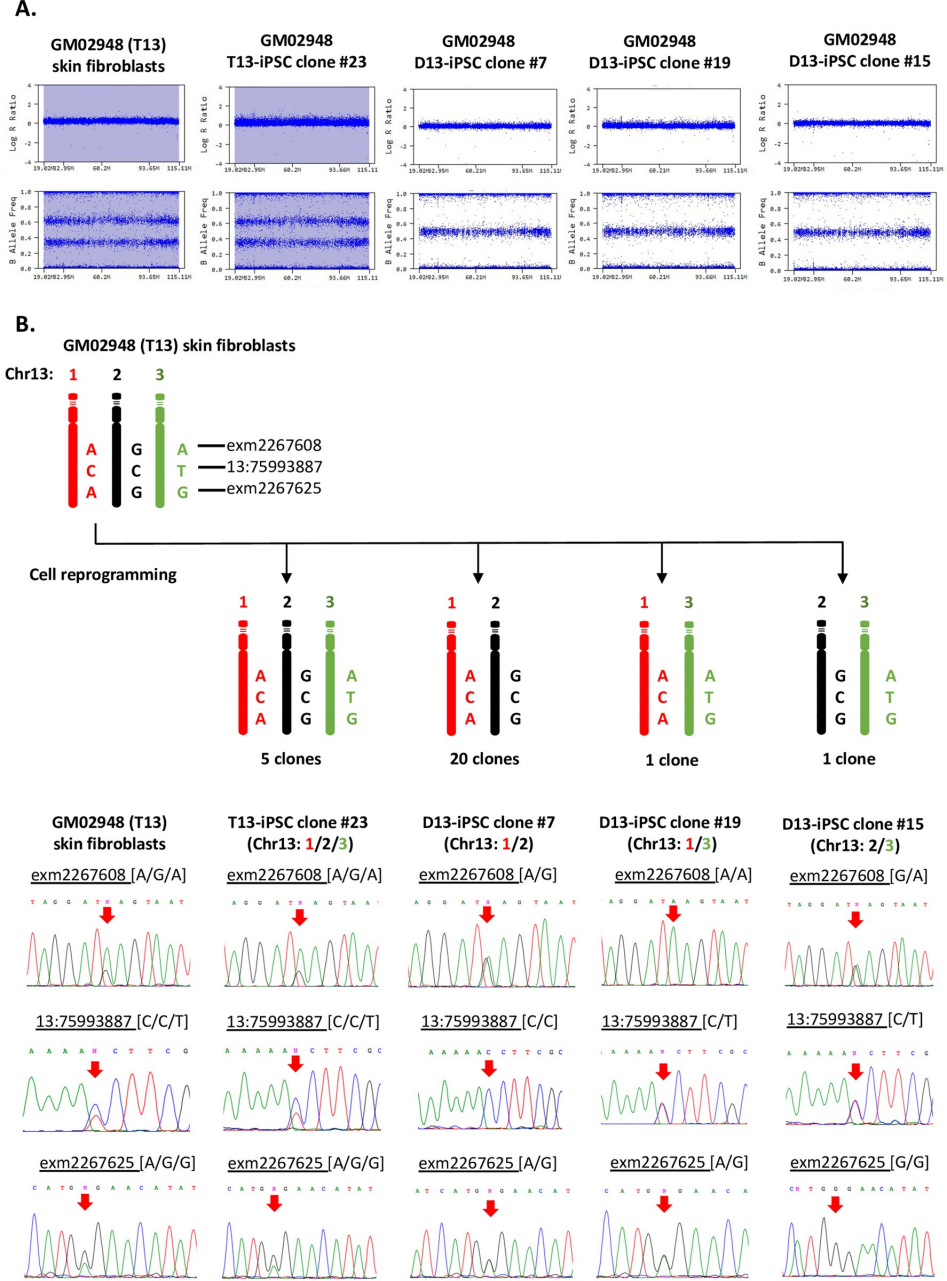
Single nucleotide polymorphism (SNP) analysis detected three different combinations of chromosome 13 pairs in the isogenic disomic iPSC clones. (A) SNP profiles of GM02948 skin fibroblasts and their isogenic iPSC clones; #23, #7, #19, and #15. The fibroblasts and the non-rescued clone #23 both show full trisomy 13, while the trisomy-rescued clones #7, #19, and #15 show heterodisomy of chromosome 13. (B) Classification of the trisomy-rescued iPSC clones into three groups by the SNP genotyping data. Three SNPs (exm2267608-rs1992744; Chr13:75993887-rs9530435, and exm2267625-rs9514690) were selected and the nucleotides were determined by Sanger sequencing. The fibroblasts and iPSC clone #23 both show SNPs of all three different chromosomes, while iPSC clones #7, #19, and #15 show SNPs of just two different chromosomes: The 1st and 2nd, 1st and 3rd, and 2nd and 3rd, respectively.

To investigate the chromosome 13 status before and after cell reprogramming in more detail, we performed single nucleotide polymorphism (SNP) genotyping analysis. The SNP profiles of chromosome 13 in skin fibroblasts (GM02948) and the five non-rescued iPSC clones both showed log R ratios of 0.5 and B allele frequency of AAA, AAB, ABB, and BBB, indicating that these cells were indeed trisomy 13. Conversely, the trisomy-rescued iPSC clones showed the log R ratios of 0.0 and B allele frequency of AA, AB, and BB, confirming disomy 13 (Fig 2A). Moreover, it was found that the trisomy-rescued iPSC clones could be classified into three groups because the SNP genotyping data allowed us to distinguish three different chromosomes, the 1st, 2nd, and 3rd, indicated in red, black, and green, respectively (Fig 2B). We selected three SNPs (rs1992744, rs9530435, and s9514690) and determined the nucleotides by Sanger sequencing to quantitatively validate the SNPs array data (Fig 2B). It was found that 20 out of the 22 trisomy-rescued iPSC clones had a combination of the 1st and 2nd chromosomes, while one iPSC clone (iPSC#19) had the 1st and 3rd chromosomes, and another iPSC clone (iPSC#15) had the 2nd and 3rd chromosomes. All three combinations showed heterodisomy in their SNP profile (Fig 2A), suggesting that the patient’s trisomy may have resulted from chromosome non-disjunction without crossing over at meiosis I in one of his parents.

To eliminate the possibility of contamination with other iPSCs, short tandem repeat (STR) analysis was performed on GM02948 fibroblasts, two clones of non-rescued iPSCs (iPSC#23 and #25), and seven clones of trisomy-rescued iPSCs (iPSC#7, #10, #5, #18, #21, #19, and #15) (Table 2). The parental fibroblasts and the two non-rescued iPSC clones showed three polymorphisms at the D13S317 locus on chromosome 13. In contrast, the seven trisomy-rescued iPSC clones lost one of the polymorphisms, which was consistent with the chromosome loss identified by the above SNP analysis (Fig 2). The STR analysis of the other chromosomes matched exactly in all cells analyzed. These results indicated that the iPSC clones established were derived from the parental GM02948 skin fibroblasts.

**Table 2:** Short tandem repeat (STR) analysis of skin fibroblasts GM02948 and their isogenic iPSC clones.

Locus	skin fibroblast P5 (T13)			T13-iPSC#23			T13-iPSC#25			D13-iPSC#7		D13-iPSC#10		D13-iPSC#5		D13-iPSC#18		D13-iPSC#21		D13-iPSC#19		D13-iPSC#15	
																							
D3S1358	16	17		16	17		16	17		16	17	16	17	16	17	16	17	16	17	16	17	16	17
TH01	6	8		6	8		6	8		6	8	6	8	6	8	6	8	6	8	6	8	6	8
D21S11	28	29		28	29		28	29		28	29	28	29	28	29	28	29	28	29	28	29	28	29
D18S51	12	14		12	14		12	14		12	14	12	14	12	14	12	14	12	14	12	14	12	14
Penta_E	7	12		7	12		7	12		7	12	7	12	7	12	7	12	7	12	7	12	7	12
D5S818	11			11			11			11		11		11		11		11		11		11	
D13S317	10	11	13	10	11	13	10	11	13	11	13	11	13	11	13	11	13	11	13	10	13	10	11
D7S820	9	11		9	11		9	11		9	11	9	11	9	11	9	11	9	11	9	11	9	11
D16S539	12	13		12	13		12	13		12	13	12	13	12	13	12	13	12	13	12	13	12	13
CSF1PO	11	12		11	12		11	12		11	12	11	12	11	12	11	12	11	12	11	12	11	12
Penta_D	10			10			10			10		10		10		10		10		10		10	
AMEL	X	Y		X	Y		X	Y		X	Y	X	Y	X	Y	X	Y	X	Y	X	Y	X	Y
vWA	14	15		14	15		14	15		14	15	14	15	14	15	14	15	14	15	14	15	14	15
D8S1179	13	15		13	15		13	15		13	15	13	15	13	15	13	15	13	15	13	15	13	15
TPOX	8	11		8	11		8	11		8	11	8	11	8	11	8	11	8	11	8	11	8	11
FGA	22	25		22	25		22	25		22	25	22	25	22	25	22	25	22	25	22	25	22	25

It has been reported that trisomy correction also occurs in late passages of trisomy 21 iPSCs -. Therefore, we cultured two clones of non-rescued GM02948 iPSCs (iPSC#24 and #25) to P10 and P8, respectively, and re-examined by karyotyping and interphase FISH. However, no karyotypic change was observed in this study (S2 Fig).

The fitness penalties of aneuploidy have been documented in many literatures [9–11]. To confirm this, we simulated the mosaicism by disomic cells co-cultured with trisomic cells. The disomic population showed an advantage over the trisomic one in both two cultures with trisomy cells: disomy cells mixture at a ratio of 9: 1 and 7: 3 (S3 Fig).

**Fig 3:**
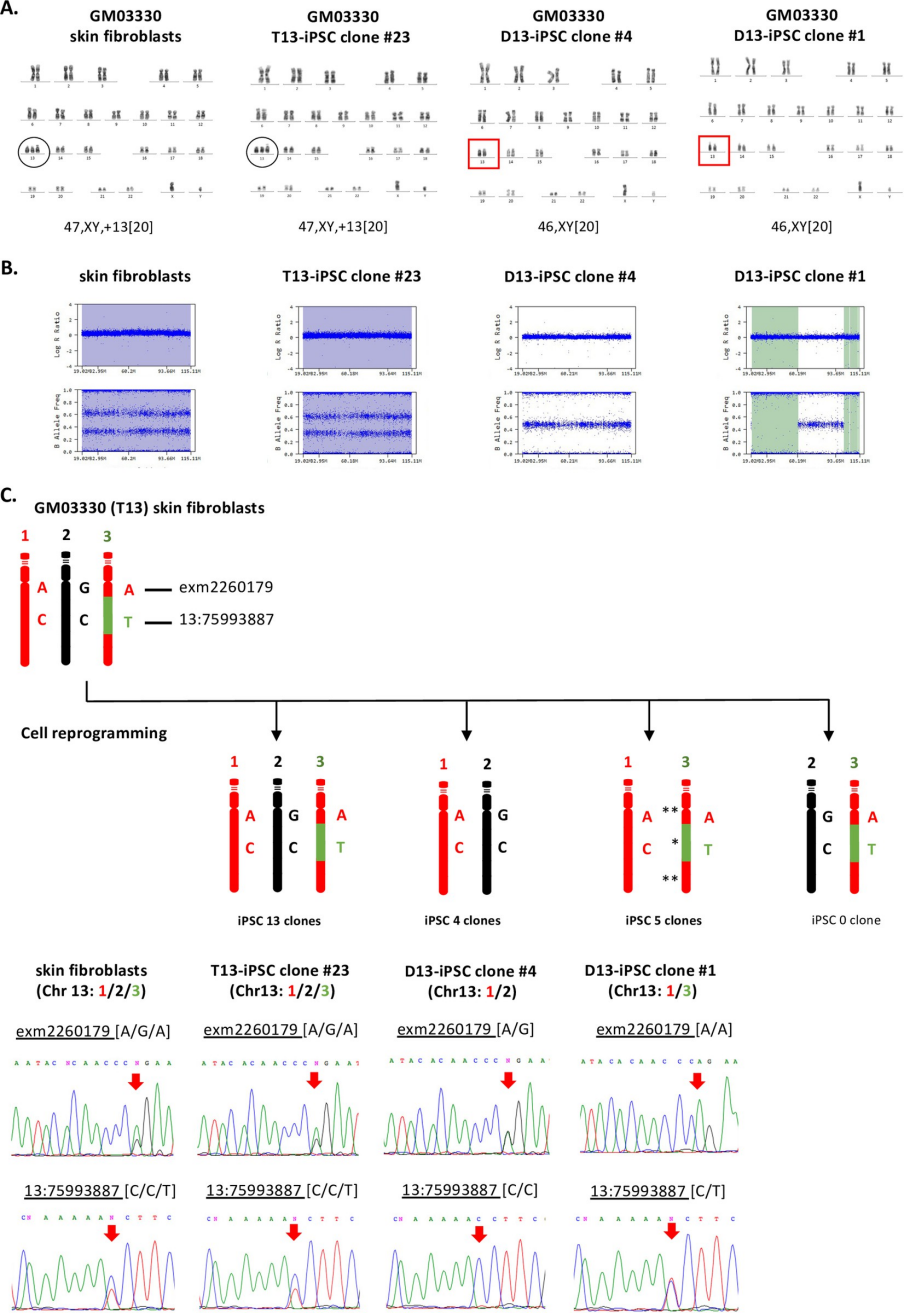
SNP and Sanger sequence indicated two combinations of chromosomes 13 pairs in GM03330 disomy iPSC clones. (A) Karyotype analysis of skin fibroblasts and the iPSC clone #23 both showed male trisomy 13. The iPSC clones #4 and #1 both showed trisomy-rescued disomy 13. (B) SNP analysis of skin fibroblasts P3 and the iPSC clone #23 both showed trisomy. In contrast, the iPSC clone #4 showed heterodisomy, and the iPSC clone #1 showed two segments of isodisomy (indicated in light green). (C) Sanger sequencing assessment of two different SNPs located on chromosome 13 (exm2260179- rs9510171, and Chr13:75993887- rs9530435) indicated a combination of the 1st and 2nd chromosomes in four iPSC clones; and a combination of the 1st and 3rd chromosomes in five iPSC clones. The UPiD is indicated by (**) and heterodisomy (*).

Next, we generated 22 iPSC clones from GM03330 skin fibroblasts by cell reprogramming using episomal vectors. Interphase FISH and karyotype analysis revealed that 9 of the 22 iPSC clones (41%) had disomy 13 (S4A Fig and Fig 3A). SNP array data and Sanger sequencing of two SNPs (rs9510171 and rs9530435) classified the nine trisomy-rescued iPSC clones into two groups, as the SNPs genotyping data allowed us to distinguish three different chromosomes: the 1st and 2nd, indicated respectively by red and black colors, and the 3rd chromosome with segments in red color (indicated by **) and one segment in green color (indicated by *). Four clones had a combination of the 1st and 2nd chromosomes, and five clones had a combination of the 1st and 3rd chromosomes. No iPSC clones with a combination of the 2nd and 3rd chromosomes were identified (Fig 3C, Table 3). The SNP array also indicated that the four clones with a combination of the 1st and 2nd chromosomes showed heterodisomy, while the five clones with a combination of the 1st and 3rd chromosomes showed two segments of uniparental isodisomy (UPiD) (indicated by **) at centromeric and distal sites (Fig 3B and 3C), suggesting that the patient’s trisomy may be a result of non-disjunction at meiosis II in one of his parents. Additional chromosomal aberrations were identified in the non-rescued iPSC clones: chromosome 11q aberration in four clones; and trisomy 20, trisomy 1, and XXY/XY mosaicism in one iPSC clone each (S4A Fig). Furthermore, SNP profiling revealed that one of the non-rescued iPSC clones with chromosome 11q aberration, iPSC#21, had whole chromosome isodisomy of chromosome 1, in addition to a reciprocal translocation between chromosomes 14 and 22. Chromosome 11q aberration was also found in a trisomy-rescued iPSC clone.

**Table 3:** Chromosome-pair selection in trisomy-rescued iPSC clones.

		Total number of trisomy-rescued iPSC clones	Number of clones with chromosome pair		
Subject	Cell line		1st and 2nd	1st and 3rd	2nd and 3rd
Trisomy 13	GM02948	22^a^	19	1	1
	GM03330	9	4	5*	-
	GM00526	5	-	-	5
Trisomy 21	GM02767	1	-	-	1
Trisomy 18	GM03538	4	1	2	1*
Trisomy 9	GM09286	15	5	6	4*

^a^A single clone with mosaic trisomy was excluded in this analysis.

*Clones with segmental uniparental isodisomy.

A skin fibroblast line from a patient with trisomy 13 (GM00526) was reprogrammed to generate 21 iPSC clones. Interphase FISH and karyotype analysis demonstrated that 5 out of the 21 iPSC lines (23.8%) had disomy 13 (S4B and S5A Figs). SNP genotyping revealed that the five trisomy-rescued iPSC clones had only one combination of the 2nd and 3rd chromosomes, which showed heterodisomy (S5B and S5C Fig). Chromosome 11q aberration was found in a trisomy-rescued clone (iPSC#1) (S4B Fig).

### Trisomy 21 rescue to disomy

**Fig 4:**
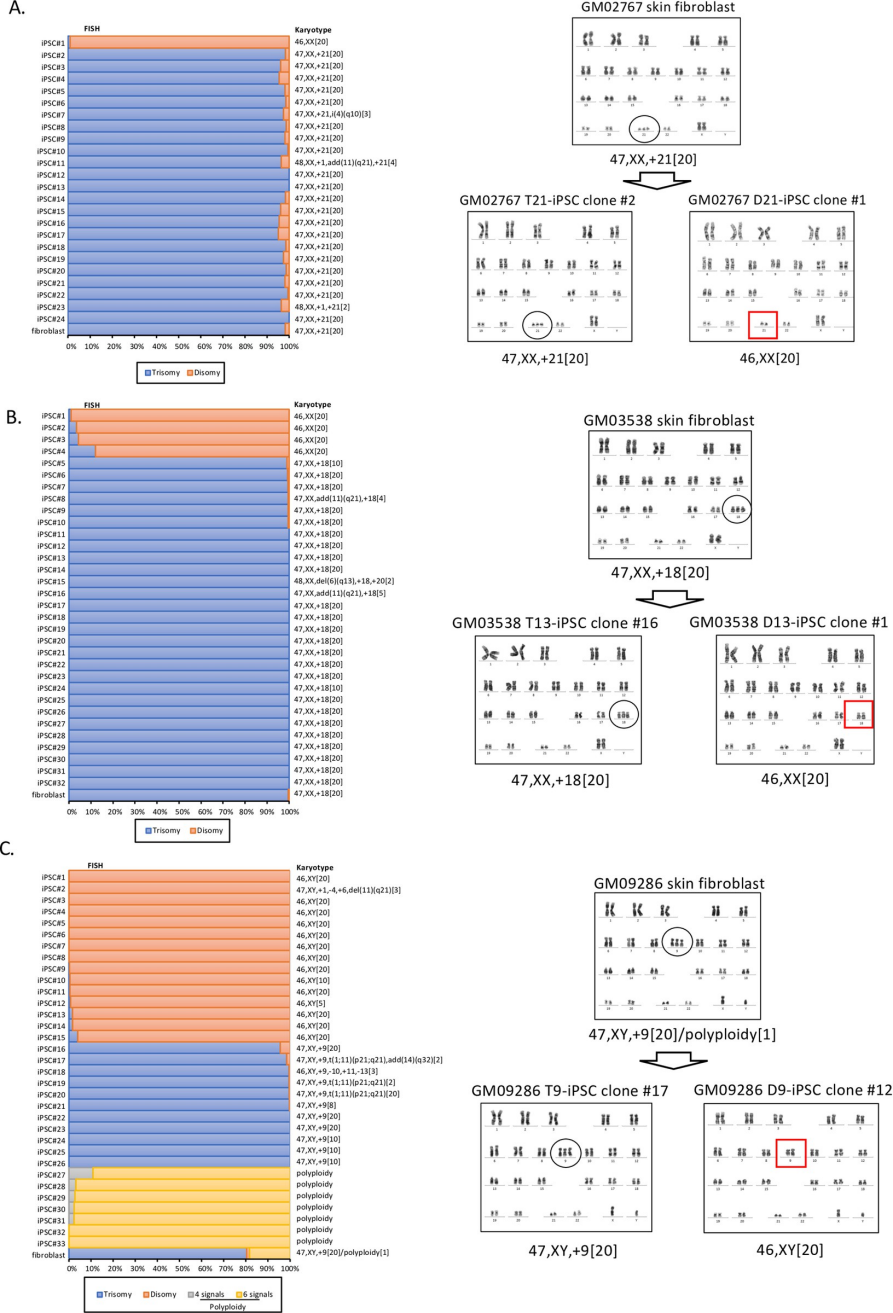
Trisomy rescue of chromosomes 21, 18, and 9 was detected by FISH and confirmed by karyotyping. (A) The left graph shows the interphase FISH results of trisomy and disomy in skin fibroblasts from a Down syndrome (DS) patient (GM02767) and 24 isogenic iPSCs, with karyotype results on the right side of the graph. Trisomy 21 is shown in blue and disomy 21 in orange. The right side shows the karyotype results of the GM02767 skin fibroblasts, the non-rescued iPSC clone #2, and the trisomy-rescued iPSC clone #1. (B) The left graph shows the FISH results of GM03638 skin fibroblasts from a patient with Edwards syndrome and 32 iPSC clones (trisomy shown in blue and disomy in orange). The right side shows the karyotype results of the GM03538 fibroblasts, the non-rescued iPSC clone #16, and the trisomy-rescued iPSC clone #1. (C) The left graph shows the FISH results of GM09286 skin fibroblasts from a patient with trisomy 9 syndrome and 33 iPSC clones (trisomy shown in blue, disomy in orange, polyploidy: Four spots in gray and six spots in yellow). The right side shows the karyotype results of the GM09286 fibroblasts, the non-rescued iPSC clone #17, and the trisomy-rescued iPSC clone #12. Polyploid cells in the GM09286 fibroblasts were not included in the karyotype results.

Skin fibroblasts were obtained from two unrelated patients with trisomy 21 (GM02767 and GM04616). Karyotypes of the two fibroblast lines were 47,XX,+21 in 20 out of 20 metaphases in both cases. Twenty-four iPSC lines were generated from GM02767 fibroblasts at P2 by cell reprogramming using episomal vectors. Interphase FISH and karyotype analysis revealed that one iPSC clone (4%, iPSC#1) had disomy 21, while the other 23 iPSC clones remained as trisomy 21 (Fig 4A). The SNP profile of the trisomy-rescued clone iPSC#1 revealed heterodisomy of chromosome 21 (S6A and S6B Fig). Karyotype analysis identified a chromosomal abnormality in three non-rescued iPSC clones each: isochromosome 4q, trisomy 1 plus an additional marker at chromosome 11q, and trisomy 1 (Fig 4A). For another fibroblast line (GM04616), we established a total of 10 iPSC clones. However, trisomy 21 rescue was not observed in any of the iPSC clones established (Table 1, S4C Fig). The two clones of non-rescued GM02767 iPSCs (iPSC#2 and #3) were further cultured to P30 and P40, respectively, and re-examined by karyotyping and interphase FISH. No karyotypic change was observed (S7 Fig).

### Trisomy 18 rescue to disomy

We obtained skin fibroblast lines from two unrelated patients with trisomy 18 (GM03538 and GM00734). Karyotype analysis of the fibroblasts revealed 47,XX,+18 in 20 out of 20 metaphases in both cases. Thirty-two iPSC clones were generated from the GM03538 fibroblasts at P2 by cell reprogramming using episomal vectors. Interphase FISH and karyotype analysis revealed that 4 out of the 32 iPSC clones (12.5%) had disomy 18 (Fig 4B).

SNPs genotyping data allowed us to distinguish three distinct copies of chromosomes 18: the 1st and 3rd chromosomes are indicated in purple and yellow, respectively, while the 2nd chromosome is shown in blue with a segment in yellow (S8B Fig). The SNP array also indicated that an iPSC clone with a combination of the 1st and 2nd chromosomes and two iPSC clones with a combination of the 1st and 3rd chromosomes all showed heterodisomy, while an iPSC clone with a combination of the 2nd and 3rd chromosomes showed segmental uniparental isodisomy (UPiD) around the centromere (indicated by **, S8A and S8B Fig). These results suggested that the patient’s trisomy may be a result of non-disjunction at meiosis II in one of her parents. Additional chromosomal aberrations were found in three non-rescued iPSC clones: trisomy 20 plus 6q deletion in one clone and an additional marker at 11q in two clones (Fig 4B).

Next, we established a total of 21 iPSC lines from GM00734 skin fibroblasts by cell reprogramming. Interphase FISH and karyotype analysis identified trisomy 13 rescue in one clone, iPSC#2 (S4D Fig). This clone was also found to show mosaicism of trisomy 13 because 38 (16%) of the 239 cells analyzed by interphase FISH showed disomy 18, whereas 201 cells still showed trisomy 18. Mosaicism was confirmed by karyotyping as having two sets of distinct metaphases (47,XX,+18[24]/46,XX[5]) (Table 1, S4D Fig).

### Trisomy 9 rescue to disomy

Full trisomy 9 is rare in live born infants, although mosaicism of trisomy 9 has been reported to be compatible with life. We obtained skin fibroblasts from a patient with trisomy 9 (GM09286). In our karyotyping of GM09286 at P5, 20 (95.2%) out of the 21 metaphases were trisomy, and polyploid cells were identified in 1 (4.8%) of the 21 cells analyzed. The average number of chromosomes in the polyploid cells was around 96. Interphase FISH showed six signals on chromosome 9 in the 54 cells (18.3%) and three signals in 237 cells (80.3%) of the 295 cells analyzed. The reason for the existence of polyploid cells is unknown, but we reprogrammed the cells at P5 to see if trisomy rescue would occur. We established a total of 33 independent iPSC clones. Interphase FISH and karyotype analysis demonstrated that 15 clones (46%) had disomy 9 while 11 clones (33%) had trisomy 9, and 7 clones (21%) showed polyploidy (Table 1, Figs 4C and S9A).

SNP array data allowed us to distinguish three distinct copies of chromosomes 9. The 1st and 3rd chromosomes are indicated in red and black, respectively. The 2nd chromosome is indicated in green (*) with two segments in black (**) and one segment in dark green (+). Sanger sequencing identified three different combinations of chromosomes 9 in the 15 rescued clones: the 1st and 2nd chromosomes in 5 clones, the 1st and 3rd in 6 clones, and the 2nd and 3rd combination in 4 clones (Table 3, S9C Fig). The SNP profiles indicated that the iPSC clones with the 1st and 2nd chromosomes and the 1st and 3rd chromosomes all showed heterodisomy, whereas the combination of the 2nd and 3rd showed segmental UPiD at both the short arm and the long arm (S9B Fig), suggesting that the patient’s trisomy may be a result of non-disjunction at meiosis I in one of his parents. Additional chromosomal aberrations were identified in 4 out of the 11 non-rescued iPSC clones and 1 out of the 15 trisomy-rescued iPSC clones (Fig 4C).

## Discussion

In this study, we found that trisomy rescue occurred upon cell reprogramming in at least one cell line with each of the trisomy syndromes 21, 18, 13, and 9, but the trisomy rescue efficiency differed greatly among the individual patients’ cells analyzed. Trisomy 21 syndrome cells showed the lowest trisomy rescue efficiencies, 4.2% for GM02967 and 0% for GM04616. Trisomy 18 syndrome cells showed efficiencies of 12.5% for GM03538 and 5.0% for GM00734. Trisomy 13 cells showed 81.5% for GM02948, 40.9% for GM03330 and 23.8% for GM00526. Trisomy 9 cells showed an efficiency of 57.7% for GM09286 when excluding the pre-existent polyploid cells (Table 1). It is currently unknown whether these differences are due to the differences in the sizes of the trisomic chromosomes, the involvement of specific genes located on the trisomic chromosome, or simply a reflection of individual cell differences.

SNP array and sequencing analyses revealed multiple combinations of chromosomes in several trisomy-rescued iPSC clones, suggesting that the selection of chromosome pairs occurs randomly. SNP array analysis also detected segmental UPiD in some of the trisomy-rescued iPSC clones, suggesting a parental origin of the trisomic chromosomes: GM03330 (T13) and GM03538 (T18) resulted from non-disjunction at meiosis II in one of the parents while GM09286 (T9) resulted from non-disjunction at meiosis I. Because either UPiD or heterodisomy was detected in the rescued iPSC clones, it seems unlikely that UPiD would have a significant impact on the efficiency of trisomy rescue. However, in cell line GM02948 (T13), trisomy rescue occurred in a biased manner (Table 3), the reason for which remains unknown.

It has been reported that reprogramming restores the karyotype not only for numerical chromosomal abnormalities but also for structural chromosomal abnormalities. Spontaneous karyotype correction was observed after reprogramming of skin fibroblasts from Miller–Dieker syndrome patients with ring chromosome 17 or 13. iPSCs generated from patient cells had lost a ring chromosome and had two intact chromosomes by dynamic mosaicism and compensatory uniparental disomy (UPD) **[12]**. Another group reprogrammed four fibroblast lines, one each with different ring chromosomes 8, 13, 18, and 22, and found spontaneous correction for ring chromosome 8 **[13]**. We established a total of 57 iPSC clones whose trisomy was rescued upon cell reprogramming. However, none of these clones had whole chromosome isodisomy for the chromosomes from which the trisomy was rescued. These results ruled out the possibility that two trisomic chromosomes were simultaneously lost and the remaining one was duplicated by the compensatory UPD mechanism, suggesting instead that one trisomic chromosome was lost to generate disomic cells upon cell reprogramming.

When does the spontaneous loss of trisomic chromosome occur? We examined the karyotypic status of iPSC clones at early passage (P2), and found that mosaicism was rarely seen. These results suggested that the loss of trisomic chromosome must occur at the very early stage of reprogramming. On the other hand, it has been reported that trisomy correction also occurs in late passages of trisomy 21 iPSCs **[8]**. However, no such karyotypic change was observed in this study (S2 and S7 Figs).

**Fig 5:**
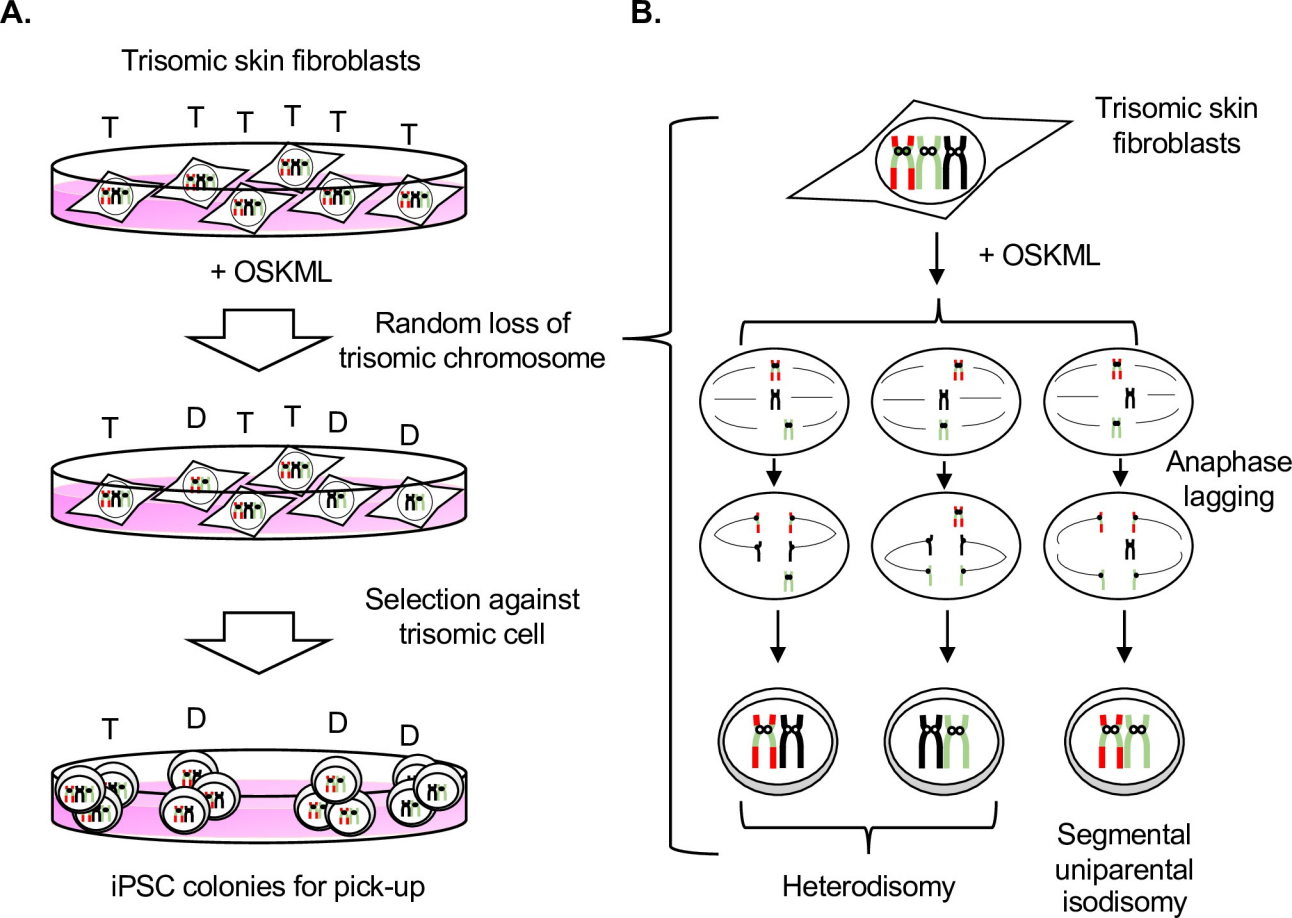
Proposed model of iPSC reprogramming-mediated trisomy rescue. (A) Random loss of trisomic chromosome occur after induction of pluripotency by expression of OSKML (Oct4, Sox2, Klf4, L-myc and Lin28) genes. Disomic cells are then selected against trisomic cells. T = trisomy; D = disomy. (B) Random loss of trisomic chromosome is mainly due to anaphase lagging.

At least two possibilities have been conceivable for the mechanisms of trisomy rescue. The first possibility is that pre-existing normal karyotype cells in the initial cell population resulting from undetectable low-level mosaicism are selected for iPSC, and the second possibility is that revertant cells (in this case, disomic) occur spontaneously and are selected for because of a growth advantage during reprogramming (Fig 5A). We have ruled out the first possibility for the following reasons: 1) multiple combinations of chromosomes were found in several trisomy-rescued iPSC clones. It seems unlikely that these cells were pre-existing in every somatic cell lines; 2) if there is a bias against reprogramming from cells with a trisomic chromosome, it would be expected that trisomic cells have reduced reprogramming efficiency. However, no such differences were observed between euploid cells and trisomic cells; and 3) two iPSC lines with trisomy mosaicism (GM02948 iPSC#22 and GM00734 iPSC#2) were identified, suggesting that the trisomic cells were reprogrammed and rescued to disomy. Regarding the second possibility, unlike somatic cells, cellular stress associated with reprogramming, such as high proliferation and weak cell cycle checkpoints, may cause the loss of the extra trisomic chromosome by random non-disjunction and then select for disomic cells because of a growth advantage. We support the second possibility for the following reasons: 1) it has been shown in many literatures that the presence of extra chromosome has a negative impact of cell fitness *in vivo* and *in vitro* **[9,10,14,15];** 2) we confirmed that disomic cells become majority over the passages in mixed cell culture (S3 Fig); and 3) non-disjunction leading to chromosomal trisomy is the most reported karyotypic change in cultured human iPSCs **[16]**. In this study, we identified iPSC clones with chromosome numerical abnormalities, such as trisomies 1, 6, 11, 20, and XXY and monosomies 4, 10, and 13, and the most prevalent chromosome numerical abnormality was trisomy 1, where genes involved in cell survival, proliferation, and differentiation are present **[17,18]**. Moreover, trisomy 20 was reported to show a selective growth advantage by inhibiting apoptosis with the BCL2L1 gene as a driver **[19]**. However, despite the random non-disjunction that had occurred, the number of the trisomy-rescued clones (57 out of 190 iPSC clones) was much higher than that of the iPSC clones with the chromosome numerical abnormalities (8 out of 190 clones) (Table 2). This prompted us to consider another possibility that a revertant occurs mainly due to anaphase lagging. When anaphase lagging occurs in trisomic chromosomes, the chromosome is lost and the resulting disomic cell is selected. If this phenomenon occurs on non-trisomic chromosomes, the chromosome is lost and the resulting monosomic cell is eliminated because of a growth disadvantage or rescued to disomy by compensatory UPD mechanism. Indeed, we observed whole chromosome isodisomy of chromosome 1 in one iPSC clone (GM03330 iPSC#21), possibly due to chromosome loss and subsequent monosomy rescue. We speculated that this hypothesis could partially explain why chromosome loss is biased toward trisomy (Fig 5B). The phenomenon of trisomy rescue is also seen in early preimplantation embryos, and it has been reported that anaphase lag mechanism may explain random loss of the trisomic chromosomes of both maternal and paternal origin because of a four-fold excess of chromosome loss compared to chromosome gain **[20,21].** Interestingly, a study in non-invasive prenatal testing detected trisomy, UPiD, and heterodisomy mosaicism in a single pregnancy, demonstrating that random and multiple trisomy rescue events occurred during early embryogenesis **[22]**. These lines of evidence suggest that trisomy rescue during cell reprogramming is analogous to that in early embryos. Trisomy rescue in early embryos has been associated with the development of human disorders such as mosaic trisomy syndrome, imprinting diseases, and confined placental mosaicism **[23–25]**. Further study will be required to clarify the mechanism of trisomy rescue.

Several types of chromosome therapy have been developed to correct the karyotypes of patients with chromosomal abnormalities in cultured cells **[22]**. Among them, trisomy rescue by cell reprogramming does not involve genome manipulation to alter the genomic DNA. Therefore, if the ethical and technical issues are resolved, trisomy rescue by cell reprogramming may be applied at the earliest stage in future regenerative and transplantation medicine, in cases such as infertility, cancer treatment, and ex vivo cell therapy. However, there are some disadvantages to chromosome therapy by cell reprograming. The first one is that even after reprogramming, trisomy rescue may not occur in some cell lines. If trisomy-rescued clones cannot always be obtained, the potential for chromosome therapeutic application would be limited. Second, UPiD can cause homozygosity for a recessive mutation or dysregulation of an imprinted gene, which may lead to cell carcinogenesis or imprinting disease-like conditions, respectively. It is therefore necessary to monitor SNPs carefully and select the cells without UPiD. Third, additional chromosomal aberrations can be seen in the iPSCs generated **[26,27]**. Therefore, it is essential to monitor the genome integrity of the iPSC clones. In this study, structural abnormalities in the long arm of chromosome 11 were repeatedly observed in the iPSC clones, the reason for which remains unclear.

In conclusion, we have demonstrated that trisomy rescue may randomly lose one copy of the trisomic chromosomes in the parental cells, resulting in three possible combinations of chromosome pairs, and then select the euploid cells. Our study reveals a part of the complexity and potential of cell reprogramming for future use in chromosome therapy. Furthermore, because isogenic trisomy-rescued iPSCs have the same genetic background and differ only in the trisomic chromosome, they will be of use in understanding and identifying the genes responsible for the clinical features of trisomy syndromes.

## Materials and methods

All studies were approved by the Ethics Committee of Hiroshima University.

### Human skin fibroblast cell lines

GM02948 (trisomy 13), GM03330 (trisomy 13), GM00526 (trisomy 13), GM04616 (trisomy 21), GM04767 (trisomy 21), GM03538 (trisomy 18), GM00734 (trisomy 18) and GM09286 (trisomy 9) were obtained from the Coriell Institute for Medical Research. Human skin fibroblasts were cultured in DMEM (Dulbecco’s Modified Eagle’s Medium; Sigma, Cat. no. D5671) supplemented with 20% fetal bovine serum and gentamicin at 37°C in humidified air with 5% CO_2_.

### Establishment and culture of human iPSCs

Human skin fibroblast cells were reprogrammed using integration-free episomal vectors carrying Oct-4, Sox2, Lin28, L-Myc, Klf4, mp53DD, and EBNA from the Ei5^TM^ Episomal iPSC Reprogramming kit (Life Technology, Cat.no. A15960). Primary fibroblast cells were cultured at a density of 10^5^ cells, electrotransfected (Lonza, Switzerland), and seeded on plates coated with Geltrex (Life Technology, Cat.no. A1413301). Cells were cultured in N2B27 medium supplemented with 10 μM Y-27632 (Wako, 253–00513, Japan) until day 15 and then this was replaced with StemFlex medium (Life Technology, Cat.no. A1517001) until iPSC colonies appeared. The colonies were isolated and their pluripotency analyzed. The iPSCs were maintained in E8 Flex medium (Life Technology, Cat.no. A2858501) on vitronectin-coated dishes and passaged using 0.5 mM EDTA in PBS.

### Immunocytochemistry analysis of pluripotency

Human skin fibroblasts (GM02948) and the iPSC clones generated were stained for key pluripotent markers using the PSC 4-marker immunocytochemistry kit (Invitrogen, Cat.no.A24881) with the following combinations of primary and secondary antibodies: anti-Oct4 (host: rabbit) and Alexa Fluor 555 donkey anti-rabbit, anti-SSEA4 (host: mouse IgG3) and Alexa Fluor 488 goat anti-mouse IgG3, anti-SOX2 (host: rat) and Alexa Fluor 488 donkey anti-rat, and TRA-1-60 (host: mouse IgM) and Alexa Fluor 594 goat anti-mouse IgG2a. The nuclear DNA was stained with DAPI. Cells were analyzed under confocal microscope (LSM800; Carl Zeiss Microimaging Inc.).

### Chromosomal analysis

iPSCs were seeded in 35 mm dishes and incubated for 24 h in Essential 8 flex medium (Gibco Cat. no. A2858501). After 24 h incubation, 0.04 μg/mL of KaryoMAX^TM^ colcemid^TM^ solution (Gibco Cat. no. 15212012) was added to arrest in metaphase for 2 hours. The iPSCs were dissociated by 0.05% Trypsin-EDTA (Gibco Cat. no. 25300062) and treated with a hypotonic solution (0.075 M Potassium Chloride; Nacalai Cat. no. 28513–85) for 20 min, and subsequently fixed with Carnoy’s solution (three parts methanol and one part acetic acid). Slides were prepared using an air-drying device for metaphase preparation (Hanabi, ADScience Technologies, Japan). G-banding by trypsin using giemsa (GTG) was performed in aged slides. Metaphase images were scanned with 40×/oil magnification with the Metafer 4 software (MetaSystems GmbH, Germany) connected to a motorized ZEISS AxioImager M1 (ZEISS, Germany). A total of 20 metaphases were examined in each cell line using MetaSystem Ikaros software for karyotyping, and the results were described in accordance with International System for Human Cytogenetic Nomenclature (ISCN).

### Fluorescence in situ hybridization (FISH) with overnight hybridization

Slides prepared with cells fixed in Carnoy’s solution were incubated in DNA denature solution (70% formamide, 10% of 20× SSC in water) for 6 min at 73°C. The slides were transferred to 70%, 85%, and 100% ethanol, respectively, for 1 min each, at RT. Probes were prepared in FISH hybridization buffer FFPE (Agilent Technologies, Cat. no. G9410A) and denatured for 6 min at 73°C. The slides were dried on a hot plate set at 50°C. Cells were coated with probe, covered with a cover slip, and incubated overnight in a humid and dark hybridization chamber at 37°C. The next morning, the cover slip was removed, and the slides were washed in FISH wash buffer 1 (Agilent Technologies, Cat. no. G9401A) for 2 min at 37°C, then immediately washed in FISH wash buffer 2 (Agilent Technologies, Cat. no. G9402A) for 2 min at RT. The slides were allowed to air dry in the dark room and finally VECTASHIELD antifade mounting medium and a cover slip were applied. Images of interphase cells were acquired by Metafer microscopy using MetaSystems’ automated imaging platform (Altlussheim, Germany). MetaSystem Isis software was used for the FISH analysis of the interphase cells. At least 200 interphase cells were examined in each cell line.

The probes used were as follows: SureFISH Chr9 CEP 367kb green (Agilent Technologies, Cat. no. G101069G-8), SureFISH Chr13 CEP 454kb orange-red (G101070R-8), SureFISH Chr18 CEP 711kb orange-red (G101074R-8), SureFISH Chr21 CEP 653kb green (G101087G-8).

### DNA extraction for STR, aCGH and SNP analysis

Genomic DNA extraction was performed using a Blood & Cell Culture DNA Mini Kit (Qiagen, Cat. no. 13323).

### Short tandem repeat (STR) analysis

STR profiling with amplification of 16 variable microsatellite regions using the PowerPlex 16 system was performed by BEX CO., LTD.

### Array comparative genomic hybridization (aCGH)

aCGH using Agilent SurePrint G3 ISCA v2 CGH+SNP 4x180K Human Microarray was conducted by Macrogen Company. GM02948 skin fibroblasts and iPSC clone #15 were analyzed, and a normal individual was used as control DNA.

### Single-nucleotide polymorphism (SNP) genotyping array and Sanger sequencing analysis

An SNP genotyping array using an Illumina Infinitum Global Screening Array (GSA)-24 BeadChip was conducted by Macrogen company. Relevant SNPs were identified in each trisomy cell lines and verified by Sanger sequencing using an Applied Biosystem 3130 Sequencer (ThermoFisher).

### Co-culture experiment

Trisomy 13 iPSCs and disomy 13 iPSCs were dissociated with StemPro accutase cell dissociation reagent (Life Technology, Cat.no. A1110501), and seeded at a density of 2 x 10^5^ cells, on plates coated with Geltrex (Life Technology, Cat.no. A1413301). Trisomy and disomy iPSCs were mixed at ratios of 0:10, 10:0, 9:1 and 7:3, respectively, and cultured during 25 days with StemFlex medium (Life Technology, Cat.no. A1517001). Every fourth or fifth day, iPSCs were dissociated, passaged, and analyzed by interphase FISH. At least 200 interphase cells were examined in each cell culture.

## Supporting information

S1 Fig Successful reprogramming to iPSC indicated by morphology and immunocytochemistry of stem cell markers. (A) Human skin fibroblasts (P2) derived from a Patau syndrome patient (GM02948), iPSC colony formation (iPSC colonies are picked-up at this timing), and iPSC clone (P1) morphologies. (B) The immunocytochemistry of four key pluripotent stem-cell markers (*Oct4*, *SSEA4 SOX2*, and *TRA-1-60*) were expressed in iPSC and not in human skin fibroblasts (P2).
(PDF)

S2 Fig FISH and karyotype results for late passage of trisomy 13 iPSC clones. (A) FISH results from two clones of non-rescued GM02948 iPSC (iPSC#24 and #25) in early passage (P2) and late passage (P10 and P8, respectively). (B) Karyotype results showed trisomy 13 in all 20 metaphases analyzed per clone in early and late passages of the two iPSC clones.
(PDF)

S3 Fig Co-culture experiment of trisomy 13 and disomy 13 iPS cells. (A) Trisomy 13 iPSCs (GM02948 iPSC#27) and disomy 13 iPSCs (GM02948 iPSC#21) were mixed and cultured in different ratios of 0:10, 10:0, 9:1 and 7:3, respectively. Every fourth or fifth day, the iPSCs were dissociated by accutase, passaged, and analyzed by interphase FISH.
(PDF)

S4 Fig FISH and karyotype results for other individuals with Trisomy 13, Trisomy 18 and Trisomy 21. (A) Results from GM03330 (Trisomy 13) skin fibroblast and the iPSC clones. (B) Results from GM00526 (Trisomy 13) skin fibroblast and the iPSC clones. (C) Results from GM04616 (Trisomy 21) skin fibroblast and the iPSC clones. (D) Results from GM00734 (Trisomy 18) skin fibroblast and the iPSC clones.
(PDF)

S5 Fig SNP and Sanger sequence indicated one combination of chromosomes 13 pairs in GM00526 disomy iPSC clones. (A) Karyotype analysis showed male trisomy 13 for skin fibroblasts and the iPSC clone #13, while normal male karyotype for the iPSC clone #5. (B) SNP analysis of skin fibroblasts P3 and the iPSC clone #5 both showed trisomy, while the iPSC clone #9 showed heterodisomy. (C) Sanger sequencing assessment of two different SNPs (exm2267608- rs1992744, and exm2267625- rs9514690) were used to demonstrate aneuploidy correction in 5 iPSC clones with a combination of the 2nd and 3rd chromosomes.
(PDF)

S6 Fig SNP and Sanger sequence indicated a single combination of chromosomes 21 pair in a GM02767 disomy iPSC clone. (A) SNP profiling of Down Syndrome skin fibroblasts P3 and the iPSC clone #2 both showed trisomy 21, while the iPSC clone #1 showed heterodisomy 21 with a small duplication (indicated in purple). (B) Sanger sequencing assessment of two different SNPs (GSA-rs112168739, and GSA-rs10482933) were used to demonstrate aneuploidy correction in the iPSC clone #1 with a combination of the 2nd and 3rd chromosomes.
(PDF)

S7 Fig FISH and karyotype results for late passage of trisomy 21 iPSC clones. (A) FISH results from two clones of non-rescued GM02767 iPSC (iPSC#2 and #3) in early passage (P2) and late passage (P30 and P40, respectively). (B) Karyotype results showed trisomy 21 in all 20 metaphases analyzed per clone in early and late passages of the two iPSC clones.
(PDF)

S8 Fig SNP and Sanger sequence indicated all three combinations of chromosomes 18 in GM03538 disomy iPSC clones. (A) SNP analysis of Edwards Syndrome skin fibroblasts and the iPSC clone #16 showed trisomy. The iPSC clones #1 and #3 both showed heterodisomy. The iPSC clone #2 showed segmental UPiD (indicated in light green). (B) Sanger sequencing assessment of three different SNPs (exm2260808-rs569629, exm1384338- rs11877062, and exm1384354- rs2298720) were used to demonstrate random selection of chromosome pairs with a combination of the 1st and 2nd chromosomes in one iPSC clone; the 1st and 3rd chromosomes in two iPSC clones, and the 2nd and 3rd chromosomes in one iPSC clone. The UPiD is indicated by (**) and heterodisomy (*).
(PDF)

S9 Fig SNP and Sanger sequence indicated all three combinations of chromosomes 9 in GM09286 disomy iPSC clones. (A) FISH analysis using chromosome 9 centromere enumeration probe (CEP 9) showed trisomy 9 (three green signals) and polyploidy (six green signals) in interphase cells of GM09286 skin fibroblasts. On the other hand, the iPSCs showed trisomy, disomy (two green signals) and polyploidy. (B) SNP analysis of trisomy 9 skin fibroblasts and the iPSC clone #17 both showed trisomy 9. The iPSC clone #10 showed heterodisomy 9 with a small duplication. The iPSC clone #1 and #6 showed heterodisomy 9 and segmental isodisomy, respectively. (C) Sanger sequencing assessment of three different SNPs (Chr9:77175017- rs965897, Chr9:127910307- rs1549314, and Chr9: 136143372- rs545971) were used to demonstrate random selection of chromosome pairs with a combination of the 1st and 2nd chromosomes in five iPSC clone, the 1st and 3rd chromosomes in six iPSC clones, and the 2nd and 3rd chromosomes in four clones. The chromosome segments of UPiD are indicated by (**), while a segment of heterodisomy is shown by (*). A small duplicated segment is shown by (+).
(PDF)

S1 Table. Primer list for SNP assessment by Sanger sequencing (XLSX)

S2 Table. Antibody used for immunocytochemistry characterization of iPSC (XLSX)
