# Supplementary figures and images for: iPSC reprogramming-mediated aneuploidy correction in autosomal trisomy syndromes
Source: PLoS One. 2022 Mar 10;17(3):e0264965. doi: 10.1371/journal.pone.0264965 (PMC8912248; doi:10.1371/journal.pone.0264965)

A.

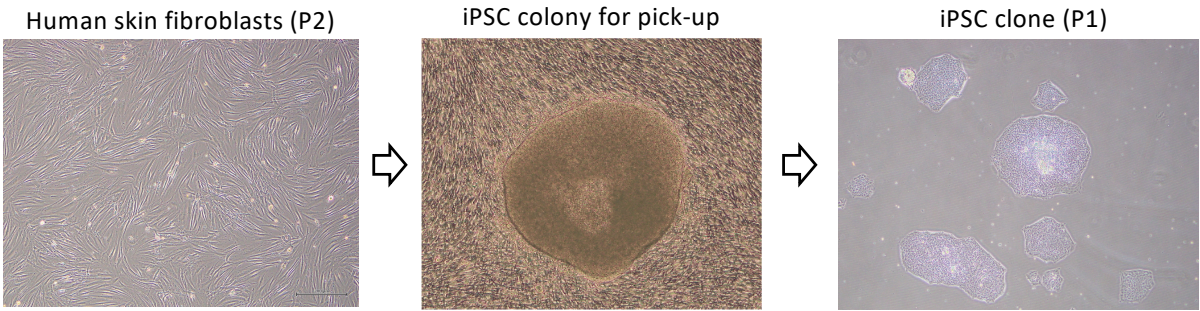

B.

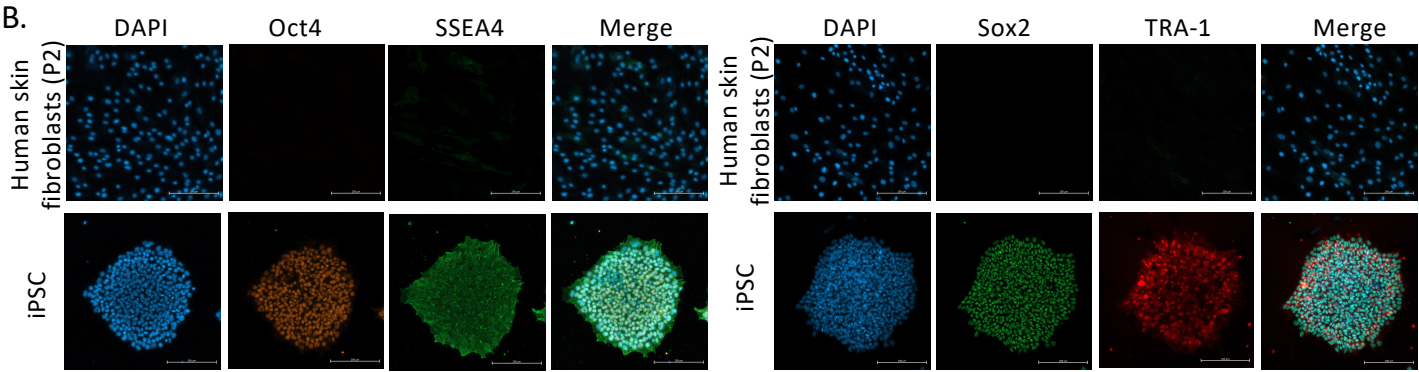

Supporting Figure 1

Supplement: S1 Fig — (A) Human skin fibroblasts (P2) derived from a Patau syndrome patient (GM02948), iPSC colony formation (iPSC colonies are picked-up at this timing), and iPSC clone (P1) morphologies. (B) The immunocytochemistry of four key pluripotent stem-cell markers (Oct4, SSEA4 SOX2, and TRA-1-60) were expressed in iPSC and not in human skin fibroblasts (P2). (PDF) [file pone.0264965.s001.pdf]

A.

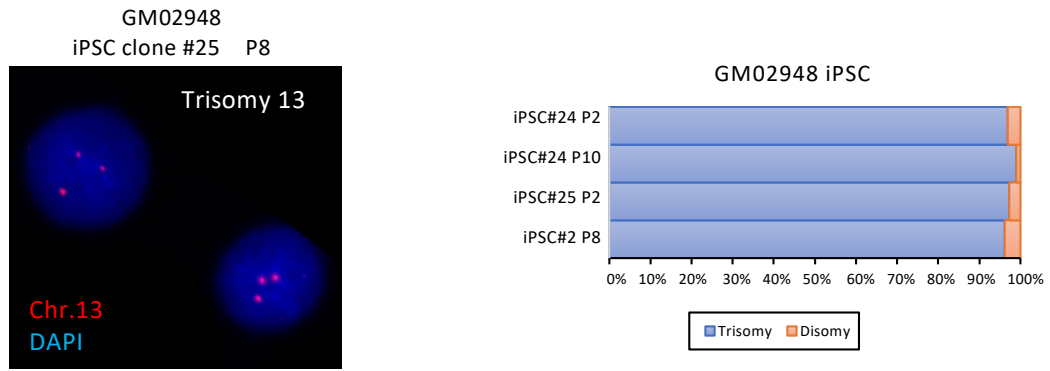

B.

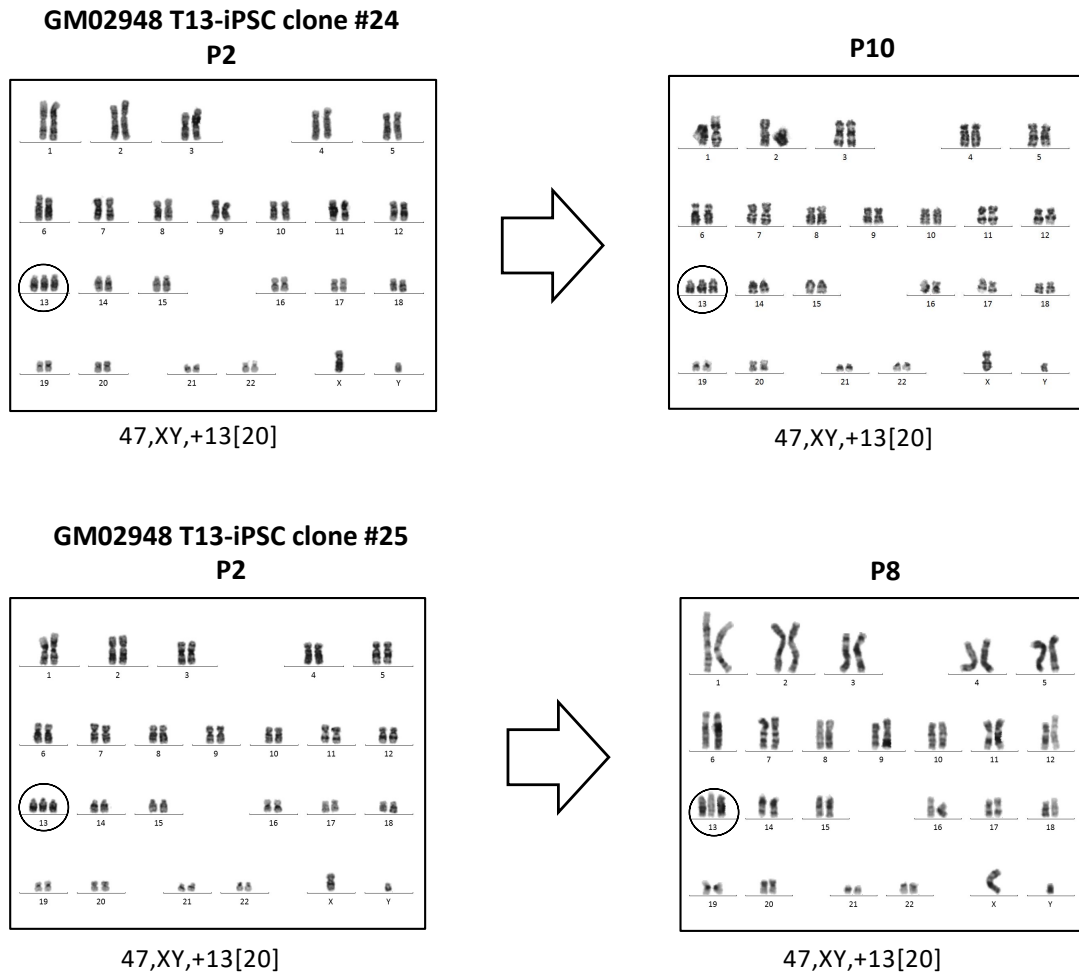

Supporting Figure 2

Supplement: S2 Fig — (A) FISH results from two clones of non-rescued GM02948 iPSC (iPSC#24 and #25) in early passage (P2) and late passage (P10 and P8, respectively). (B) Karyotype results showed trisomy 13 in all 20 metaphases analyzed per clone in early and late passages of the two iPSC clones. (PDF) [file pone.0264965.s002.pdf]

A.

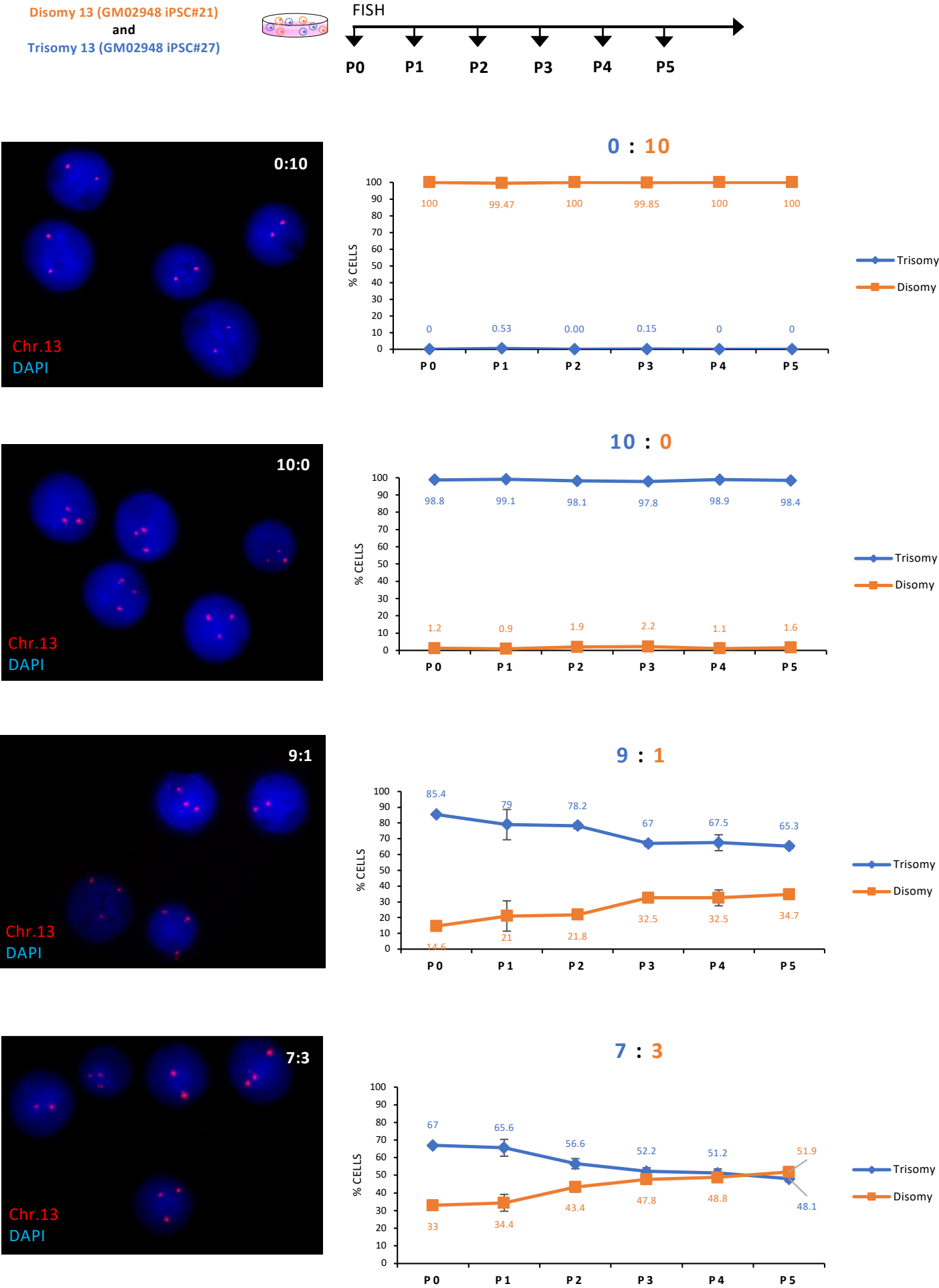

Supporting Figure 3

Supplement: S3 Fig — (A) Trisomy 13 iPSCs (GM02948 iPSC#27) and disomy 13 iPSCs (GM02948 iPSC#21) were mixed and cultured in different ratios of 0:10, 10:0, 9:1 and 7:3, respectively. Every fourth or fifth day, the iPSCs were dissociated by accutase, passaged, and analyzed by interphase FISH. (PDF) [file pone.0264965.s003.pdf]

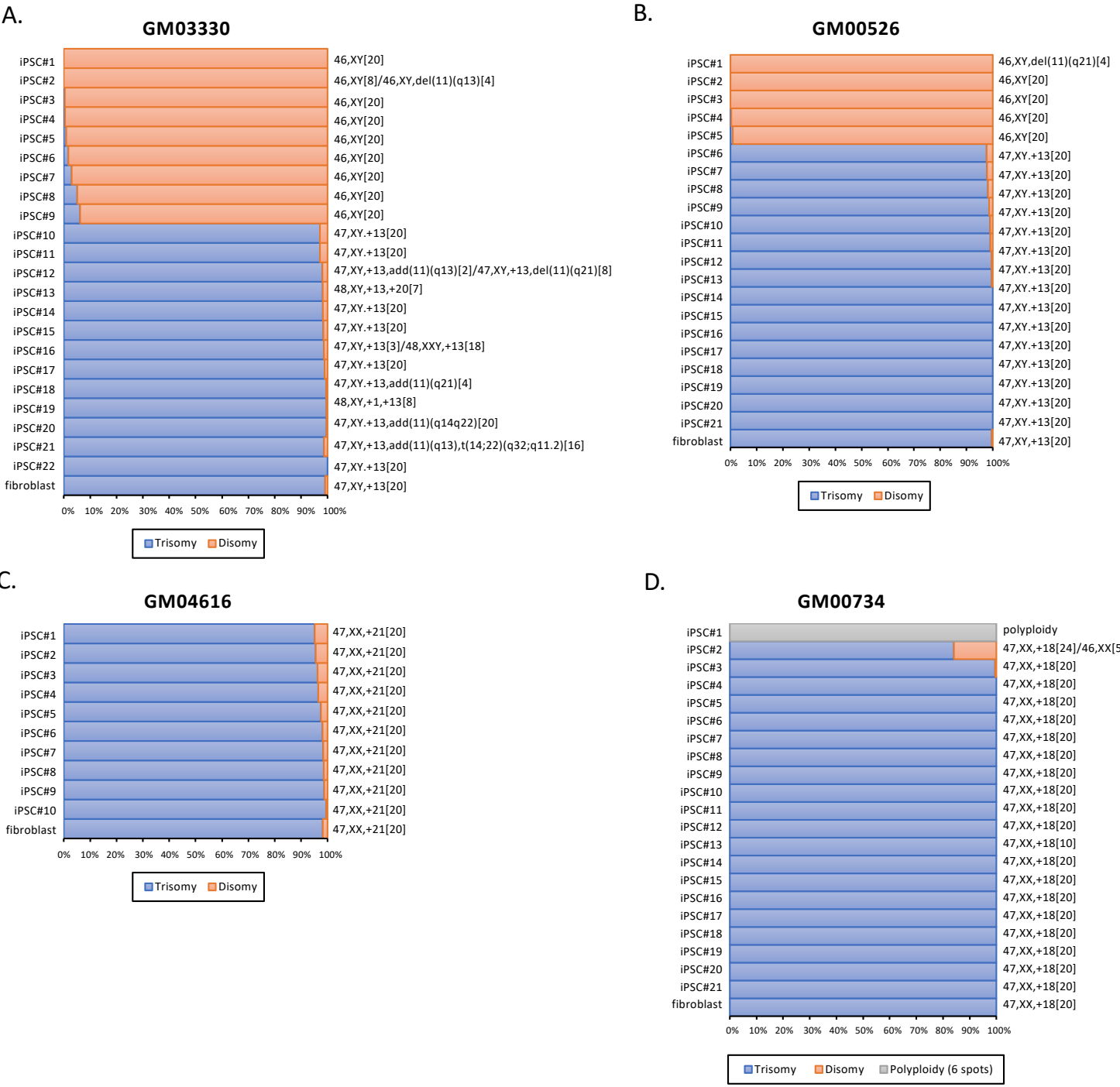

Supplement: S4 Fig — (A) Results from GM03330 (Trisomy 13) skin fibroblast and the iPSC clones. (B) Results from GM00526 (Trisomy 13) skin fibroblast and the iPSC clones. (C) Results from GM04616 (Trisomy 21) skin fibroblast and the iPSC clones. (D) Results from GM00734 (Trisomy 18) skin fibroblast and the iPSC clones. (PDF) [file pone.0264965.s004.pdf]

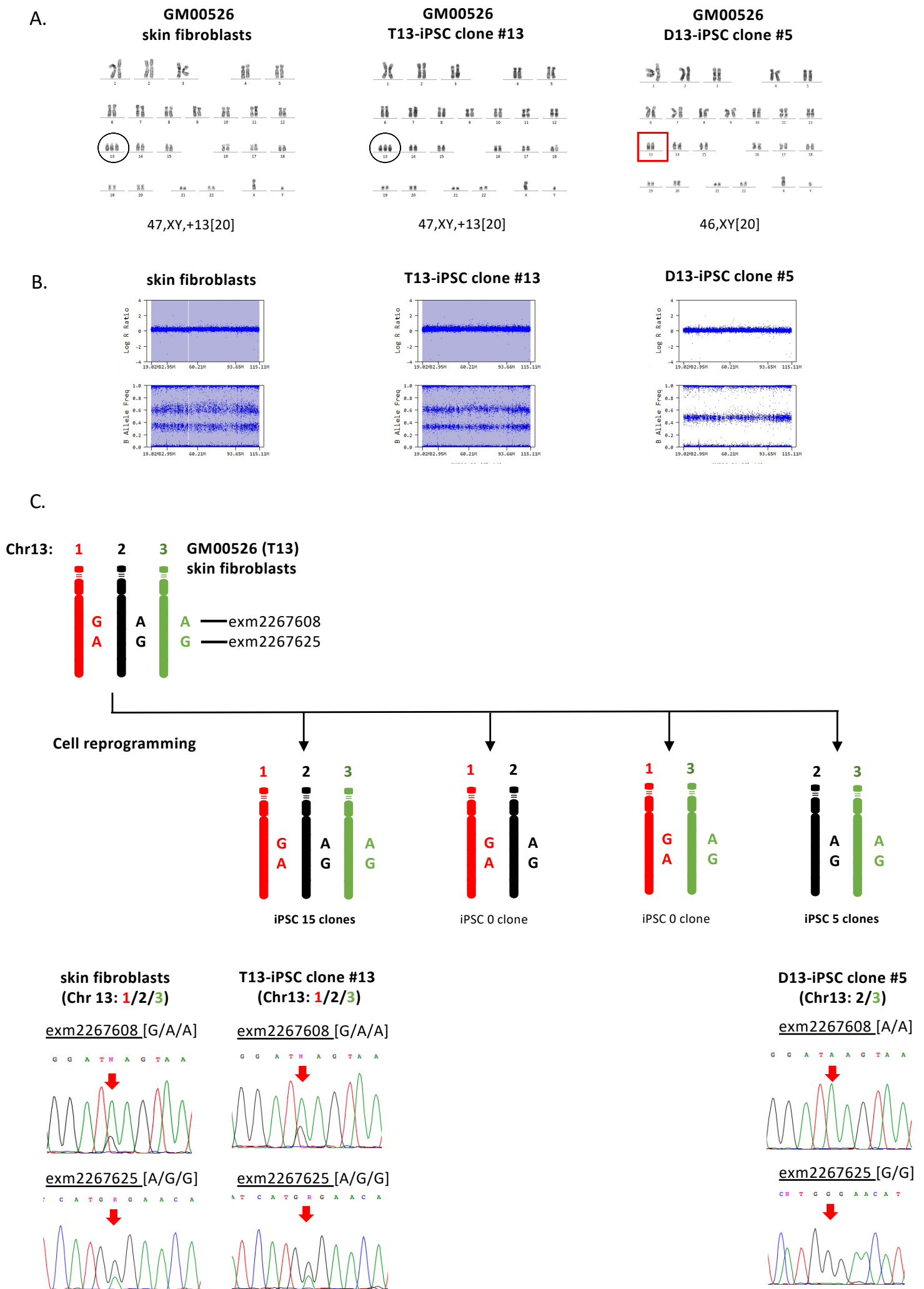

**Supporting Figure 5**

Supplement: S5 Fig — (A) Karyotype analysis showed male trisomy 13 for skin fibroblasts and the iPSC clone #13, while normal male karyotype for the iPSC clone #5. (B) SNP analysis of skin fibroblasts P3 and the iPSC clone #5 both showed trisomy, while the iPSC clone #9 showed heterodisomy. (C) Sanger sequencing assessment of two different SNPs (exm2267608- rs1992744, and exm2267625- rs9514690) were used to demonstrate aneuploidy correction in 5 iPSC clones with a combination of the 2nd and 3rd chromosomes. (PDF) [file pone.0264965.s005.pdf]

A.

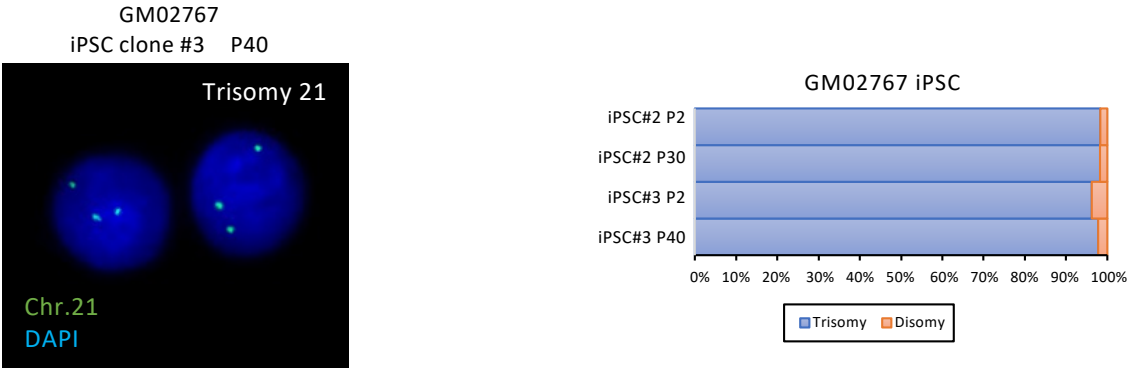

B.

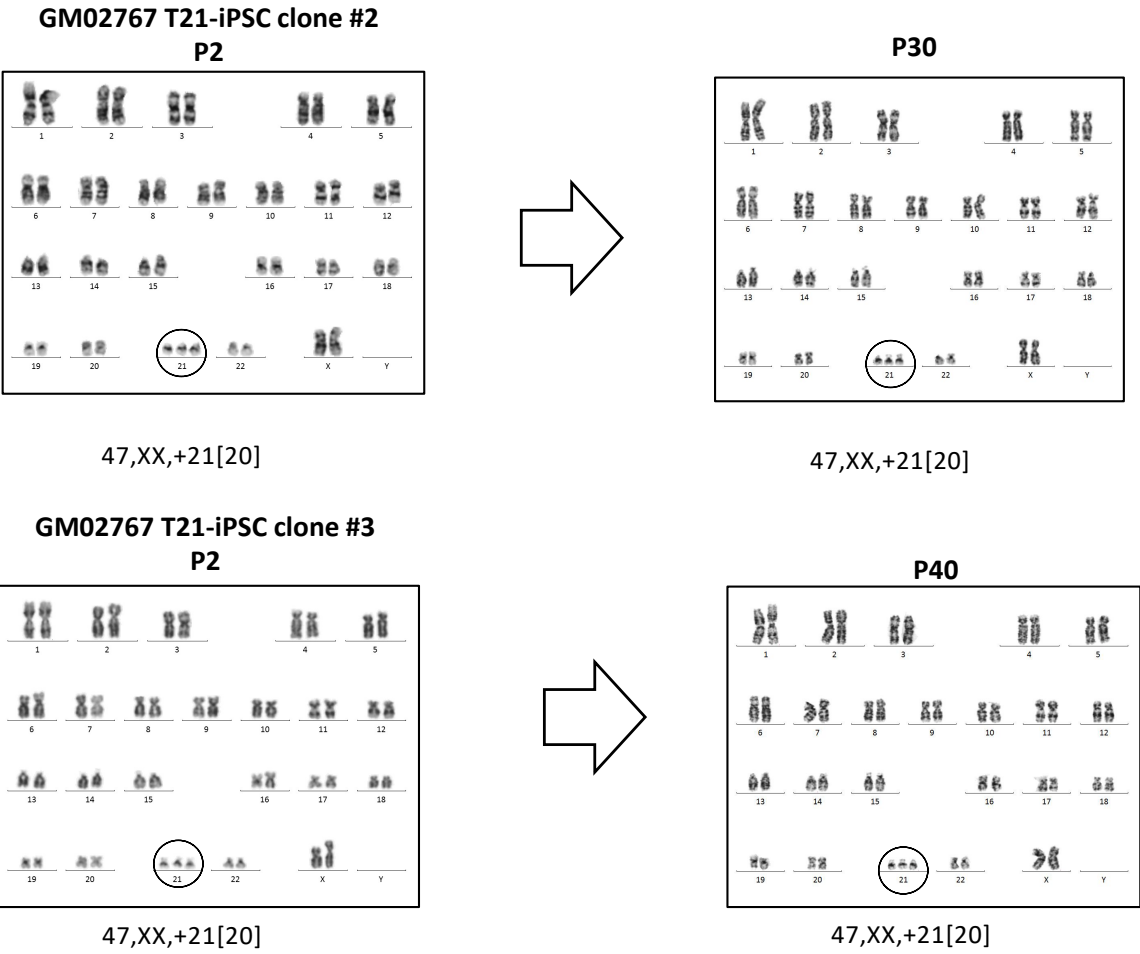

Supporting Figure 7

Supplement: S7 Fig — (A) FISH results from two clones of non-rescued GM02767 iPSC (iPSC#2 and #3) in early passage (P2) and late passage (P30 and P40, respectively). (B) Karyotype results showed trisomy 21 in all 20 metaphases analyzed per clone in early and late passages of the two iPSC clones. (PDF) [file pone.0264965.s007.pdf]

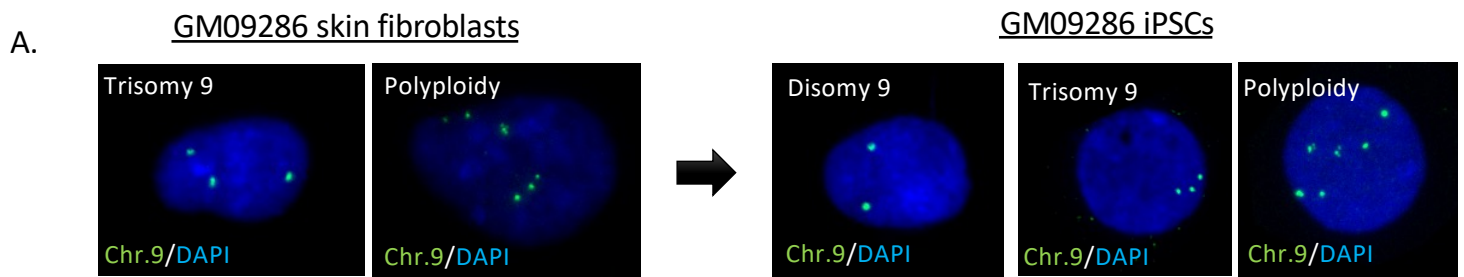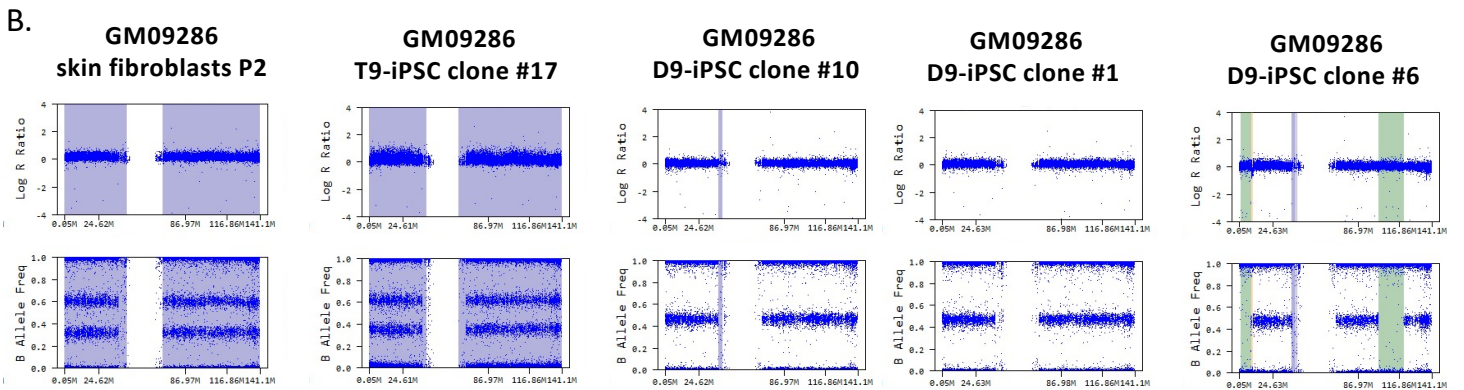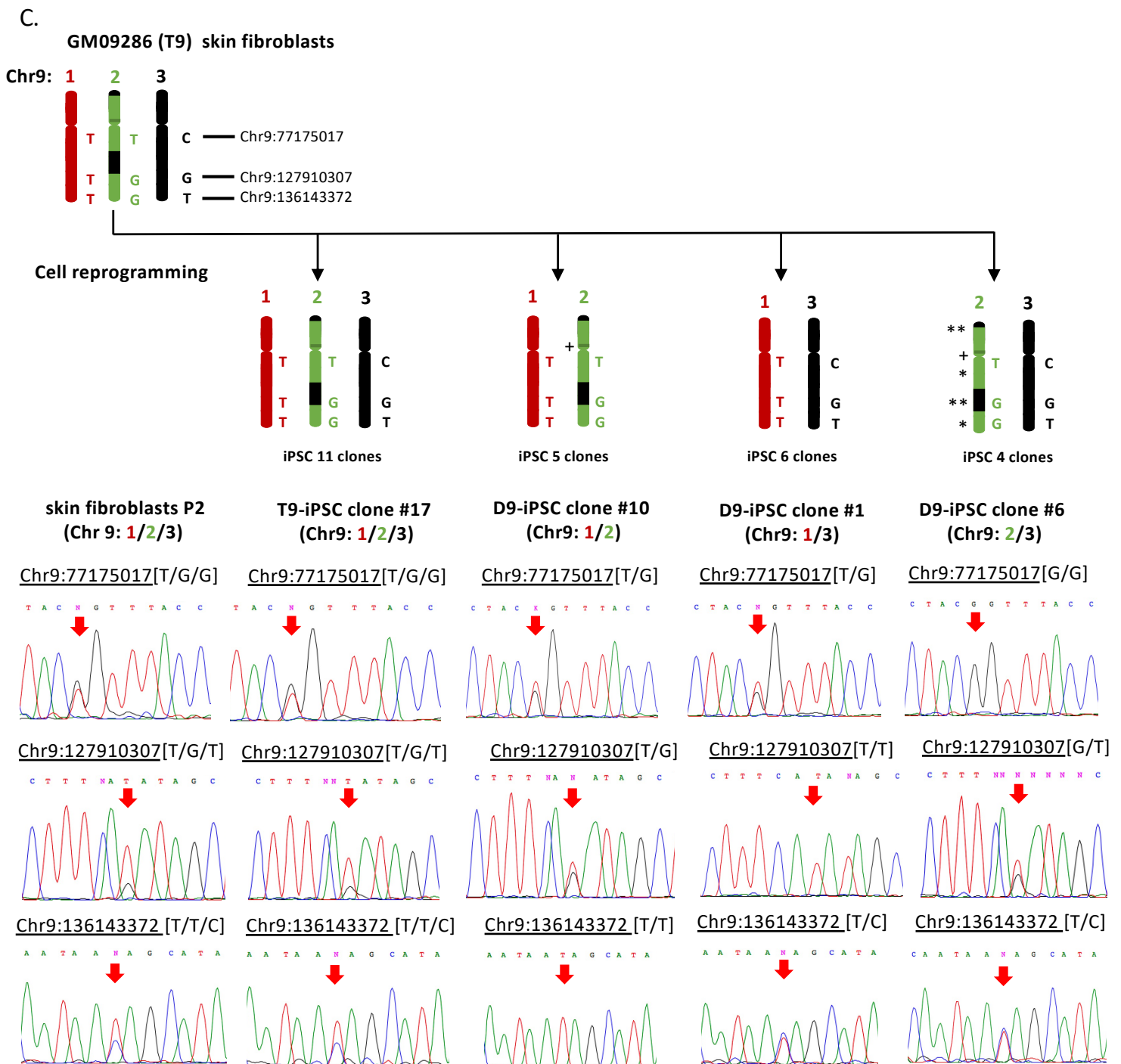

Supporting Figure 9

Supplement: S9 Fig — (A) FISH analysis using chromosome 9 centromere enumeration probe (CEP 9) showed trisomy 9 (three green signals) and polyploidy (six green signals) in interphase cells of GM09286 skin fibroblasts. On the other hand, the iPSCs showed trisomy, disomy (two green signals) and polyploidy. (B) SNP analysis of trisomy 9 skin fibroblasts and the iPSC clone #17 both showed trisomy 9. The iPSC clone #10 showed heterodisomy 9 with a small duplication. The iPSC clone #1 and #6 showed heterodisomy 9 and segmental isodisomy, respectively. (C) Sanger sequencing assessment of three different SNPs (Chr9:77175017- rs965897, Chr9:127910307- rs1549314, and Chr9: 136143372- rs545971) were used to demonstrate random selection of chromosome pairs with a combination of the 1st and 2nd chromosomes in five iPSC clone, the 1st and 3rd chromosomes in six iPSC clones, and the 2nd and 3rd chromosomes in four clones. The chromosome segments of UPiD are indicated by (**), while a segment of heterodisomy is shown by (*). A small duplicated segment is shown by (+). (PDF) [file pone.0264965.s009.pdf]
