# Supplementary material for: iPSC reprogramming-mediated aneuploidy correction in autosomal trisomy syndromes
Source: PLoS One. 2022 Mar 10;17(3):e0264965. doi: 10.1371/journal.pone.0264965 (PMC8912248; doi:10.1371/journal.pone.0264965)
Supplement: S6 Fig — (A) SNP profiling of Down Syndrome skin fibroblasts P3 and the iPSC clone #2 both showed trisomy 21, while the iPSC clone #1 showed heterodisomy 21 with a small duplication (indicated in purple). (B) Sanger sequencing assessment of two different SNPs (GSA-rs112168739, and GSA-rs10482933) were used to demonstrate aneuploidy correction in the iPSC clone #1 with a combination of the 2nd and 3rd chromosomes. (PDF) [file pone.0264965.s006.pdf]

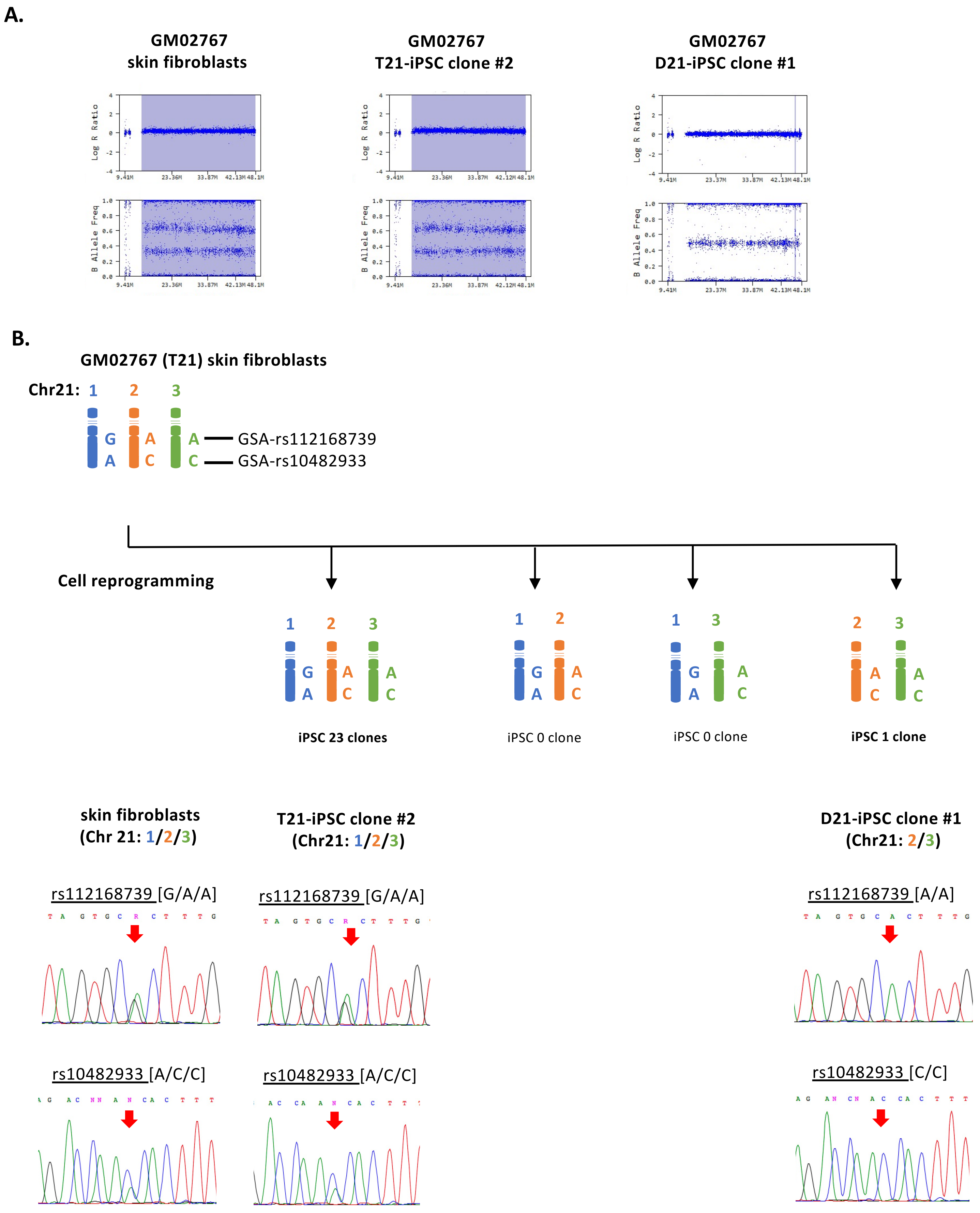

Supporting Figure 6
