# Supplementary material for: iPSC reprogramming-mediated aneuploidy correction in autosomal trisomy syndromes
Source: PLoS One. 2022 Mar 10;17(3):e0264965. doi: 10.1371/journal.pone.0264965 (PMC8912248; doi:10.1371/journal.pone.0264965)
Supplement: S8 Fig — (A) SNP analysis of Edwards Syndrome skin fibroblasts and the iPSC clone #16 showed trisomy. The iPSC clones #1 and #3 both showed heterodisomy. The iPSC clone #2 showed segmental UPiD (indicated in light green). (B) Sanger sequencing assessment of three different SNPs (exm2260808-rs569629, exm1384338- rs11877062, and exm1384354- rs2298720) were used to demonstrate random selection of chromosome pairs with a combination of the 1st and 2nd chromosomes in one iPSC clone; the 1st and 3rd chromosomes in two iPSC clones, and the 2nd and 3rd chromosomes in one iPSC clone. The UPiD is indicated by (**) and heterodisomy (*). (PDF) [file pone.0264965.s008.pdf]

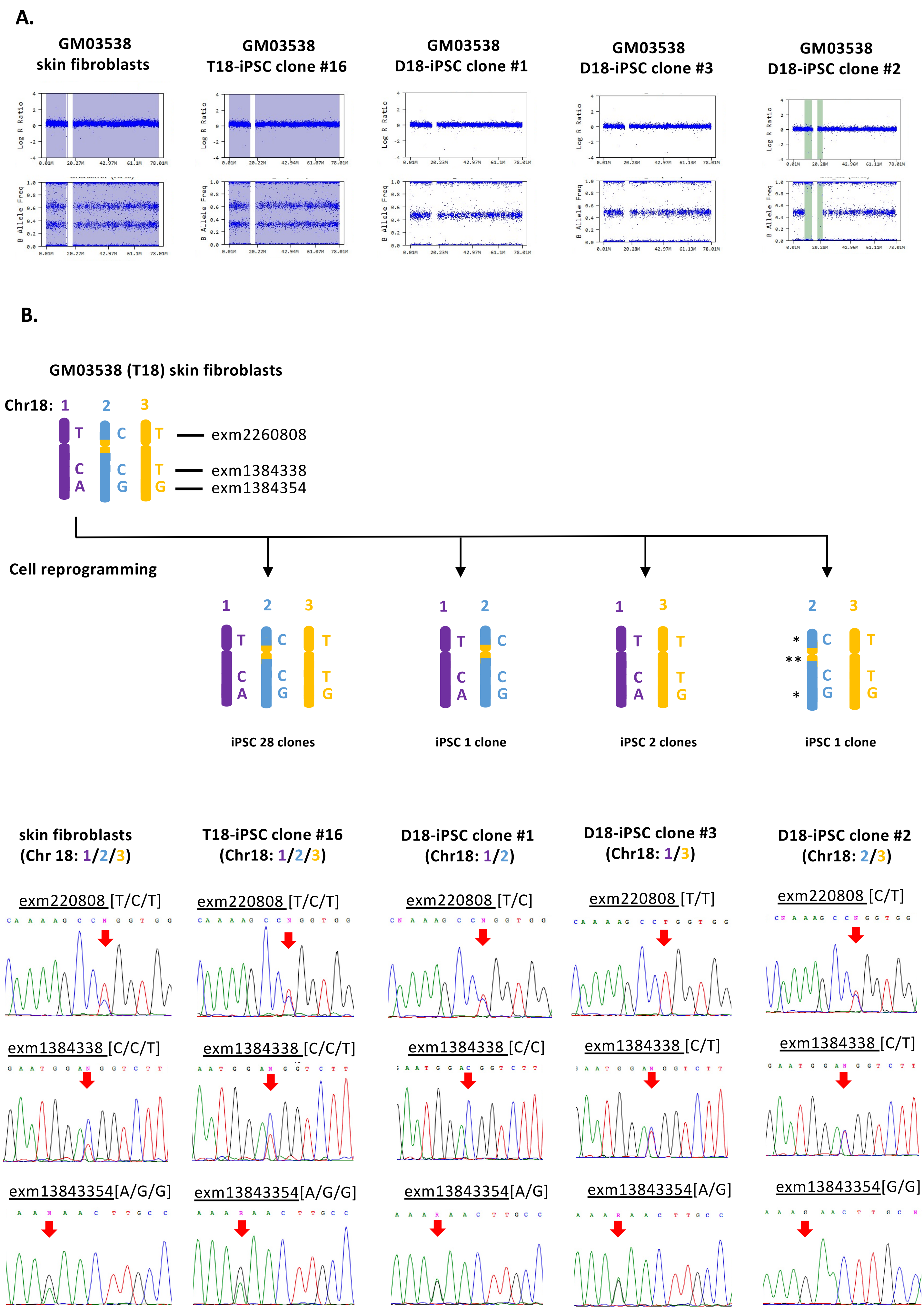

Supporting Figure 8
